# Altered proportions of retinal cell types and distinct visual codes in rodents occupying divergent ecological niches

**DOI:** 10.1016/j.cub.2025.02.014

**Published:** 2025-03-04

**Authors:** Annette E. Allen, Joshua Hahn, Rose Richardson, Andreea Pantiru, Josh Mouland, Aadhithyan Babu, Beatriz Baño-Otalora, Aboozar Monavarfeshani, Wenjun Yan, Christopher Williams, Jonathan Wynne, Jessica Rodgers, Nina Milosavljevic, Patrycja Orlowska-Feuer, Riccardo Storchi, Joshua R. Sanes, Karthik Shekhar, Robert J. Lucas

**Affiliations:** 1 Division of Neuroscience, School of Biological Sciences, Faculty of Biology, Medicine and Health, University of Manchester, Manchester M13 9PT, UK; 2 Centre for Biological Timing, Faculty of Biology Medicine and Health, University of Manchester, Manchester M13 9PT, UK; 3 Department of Chemical and Biomolecular Engineering, University of California, Berkeley, Berkeley, CA 94720, USA; 4 Division of Diabetes, Endocrinology and Gastroenterology, School of Medical Sciences, Faculty of Biology, Medicine and Health, University of Manchester, Manchester M13 9PT, UK; 5 Department of Cellular and Molecular Biology, Center for Brain Science, Harvard University, Cambridge, MA 02138, USA; 6 Helen Wills Neuroscience Institute, Vision Science Graduate Group, Center for Computational Biology, Biophysics Graduate Group, California Institute of Quantitative Biosciences (QB3), University of California, Berkeley, Berkeley, CA 94720, USA

## Abstract

Vertebrate retinas share a basic blueprint comprising 5 neuronal classes arranged according to a common wiring diagram. Yet, vision is aligned with species differences in behavior and ecology, raising the question of how evolution acts on this circuit to adjust its computational characteristics. We address that problem by comparing the thalamic visual code and retinal cell composition in closely related species occupying different niches: *Rhabdomys pumilio*, which are day-active murid rodents, and nocturnal laboratory mice (*Mus musculus*). Using high-density electrophysiological recordings, we compare visual responses at both single-unit and population levels in the thalamus of these two species. We find that *Rhabdomys* achieves a higher spatiotemporal resolution visual code through the selective expansion of information channels characterized by non-linear spatiotemporal summation. Comparative analysis of single-cell transcriptomic atlases reveals that this difference originates with the increased relative abundance of retinal bipolar and ganglion cell types supporting OFF and ON-OFF responses. These findings demonstrate that evolution may drive changes in neural computation by adjusting the proportions of shared cell types rather than inventing new types and show the power of matching high-density physiological recordings with transcriptomic cell atlases to study evolution in the brain.

## Graphical abstract

**Graphical abstract:**
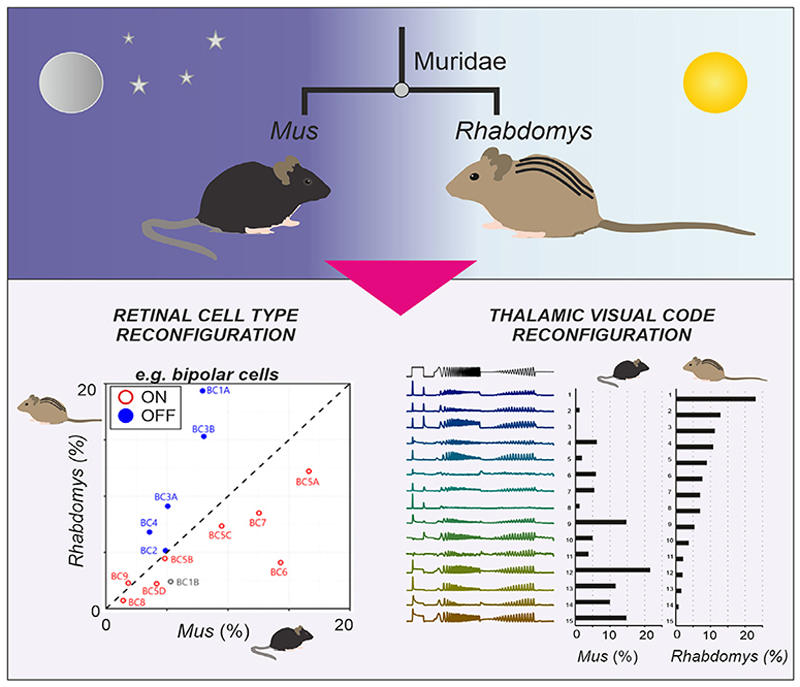

## Introduction

Vertebrate central nervous systems share common structural, developmental, and genetic blueprints. This standard architecture must accommodate species-specific differences in dominant sense(s), modes of locomotion, and strategies for food seeking, predator avoidance, and reproduction. Such profound differences in functional requirements can exist for species that are closely related by phylogeny, highlighting the need for plasticity in the common blueprint of the brain.^1^ Recent technological developments allow exploration of how such adaptations arise with unprecedented resolution. On the one hand, large-scale recordings of neuronal populations facilitate an unbiased comparison of information channels across species,^2,3^ linking coding strategies to ecological niches. On the other, high-throughput single-cell transcriptomics allows comprehensive identification of cell types and cross-species comparisons.^4–6^ With these techniques in hand, it becomes possible to describe the process of central nervous system evolution by tracing changes in cell-type complement and adaptations in computational characteristics.

Here, we apply this approach to a comparison of the early visual system in two closely related murid rodents. The laboratory mouse (*Mus musculus*) is a popular model for understanding mammalian vision in health and disease and for establishing general principles of neural function. Like most murid species, *Mus* are predominantly nocturnal, and this is reflected in key features of the visual system, including a rod dominant retina and a UV-transmitting lens.^7^ However, some Muridae are day-active, including the four-striped mouse, *Rhabdomys pumilio*, of sub-Saharan Africa.^8,9^ The switch to daytime activity in *Rhabdomys* has been associated with substantial changes in visual system anatomy.^10,11^ The *Rhabdomys* retina is cone dominated, its lens absorbs UV light, and both its retina and visual centers in the brain are expanded in volume compared with *Mus*.^10–12^ Moreover, an unbiased analysis of the *Rhabdomys* genome reveals that genes involved in vision exhibit accelerated evolution compared with related murids.^13^

*Rhabdomys* and *Mus* represent a case study in how differences in temporal niche are reflected in visual system anatomy.^14^ However, an important unanswered question is how such anatomical expansion impacts the visual code. In principle, the enhanced capacity to process visual information in *Rhabdomys* could allow the same computations to be performed with higher precision; a wider array of visual features to be extracted, describing the scene with greater granularity; or reconfigurations of the visual code toward features of particular ecological importance. Similarly, contributors to the anatomical expansion remain unclear. For example, both inner nuclear and ganglion cell layers are thicker in the *Rhabdomys* retina,^10–12^ but the increased cell number might be accompanied by altered frequencies of cell types shared with *Mus*, emergence of new types, or both. Addressing these questions will help reveal how the blueprint of neural circuits can be adjusted to align computation to changing ecology.

To address these issues, we combine high-density electrophysiological recordings of visual response properties in the dorsal lateral geniculate nucleus (dLGN; primary visual thalamus, the principal relay of information from the retina to the cerebral cortex) of *Mus* and *Rhabdomys*, with comparative analyses of retinal cell atlases from these species. We find that the enhanced capacity of the *Rhabdomys* visual system is applied not only to sample the scene with higher density but also to facilitate a realignment toward non-linear spatiotemporal summation. This functional realignment can be traced to adjustments in the relative abundance of multiple bipolar and retinal ganglion cell (RGC) types. Our data thus show how evolution can shape computation by altering the frequencies of specific cell types within the framework of a common neural circuit blueprint rather than by “inventing” new types.

## Results

### Realignment in thalamocortical visual code between *Mus* and *Rhabdomys*

**Figure 1:**
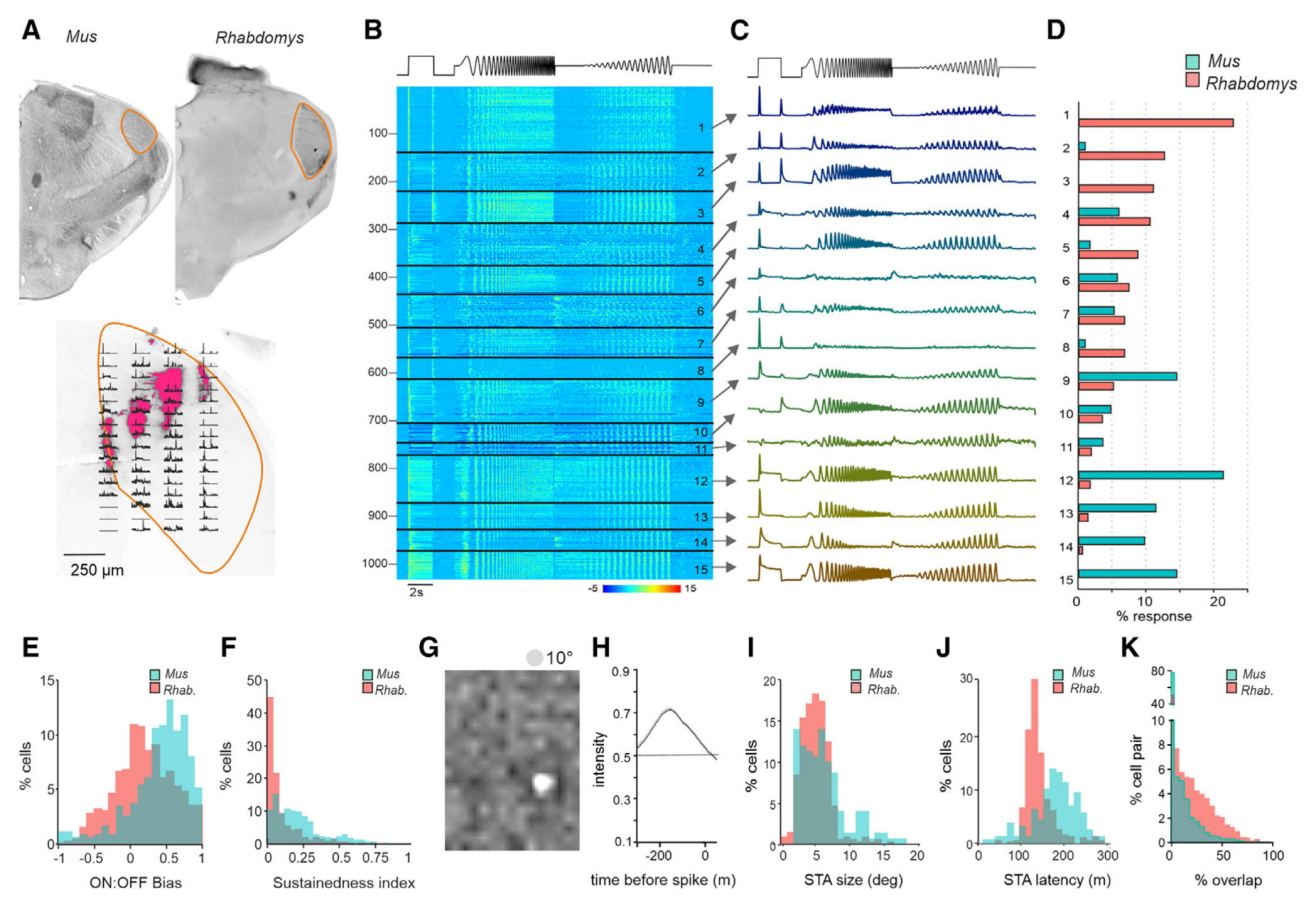
dLGN response diversity in *Mus* and *Rhabdomys* (A) Above: coronal sections from *Mus* and *Rhabdomys* with dLGN highlighted with orange edging. Below: histological reconstruction of recording sites in *Rhabdomys* dLGN. Pink shows fluorescence (DiI) markings remaining after electrode insertion. Peristimulus time histograms (PSTHs) representing multiunit light-evoked activity recorded at each recording site overlaid (2 s light step with 1 s pre and post stimulus). All data are scaled according to their maximum firing rate. (B) Heatmap showing normalized PSTHs of dLGN neurons as a function of response cluster (labeled to right), organized as a function of number of *Rhabdomys* responses. Stimulus profile denoted above. *n* = 422 and *n* = 611 units in *Mus* and *Rhabdomys*, respectively. (C) Mean response profile of normalized responses from each cluster (combined across species). Stimulus profile denoted above. (D) Bar graphs showing proportion of responses within each cluster for each species (*Mus*: cyan; *Rhabdomys*: red). (E) Histogram of ON:OFF bias in *Mus* (cyan) and *Rhabdomys* (red). (F) Histogram of sustainedness index in *Mus* (cyan) and *Rhabdomys* (red). (G and H) Example spatial (G) and temporal (H) spike-triggered averages recorded in response to binary noise stimulus. (I) Histogram of receptive field sizes in *Mus* (cyan) and *Rhabdomys* (red); Kolmogorov-Smirnov test *p* = 0.036. *n* = 346 and *n* = 419 units in *Mus* and *Rhabdomys*, respectively. (J) Histogram of receptive field latency (time of peak absolute response) in *Mus* (cyan) and *Rhabdomys* (red); Kolmogorov-Smirnov test: *p* < 0.001. (K) Histogram of receptive field overlap between pairs of neurons recorded in *Mus* (cyan) and *Rhabdomys* (red); Kolmogorov-Smirnov test: *p* < 0.001. See also Figures S1–S4 and S6.

Based on the assumption that thalamocortical vision may more reliably reflect inter-species differences in visual ecology than parts of the visual system responsible for reflexive behaviors, such as optomotor coordination, pupil regulation, and circadian entrainment, we set out to compare the visual code in *Rhabdomys* and *Mus* dLGN. Using a 256-channel recording electrode across multiple placements in both sagittal and coronal orientations, we recorded visually evoked activity for neurons across the dLGN of both species (Figures 1A and S1). To broadly survey the diversity of visual responses in the two species, we applied a full-field temporal modulation stimulus (Figure 1B) previously used in both the retina and dLGN of *Mus* to identify functional response types.^15^ dLGN neurons in anesthetized animals of both species showed strong yet diverse responses to this stimulus (*n* = 611 and *n* = 422 units in *Rhabdomys* and *Mus*, respectively; Figure 1).

To explore the characteristics and numbers of response types within each species, we pooled light-responsive units from *Rhabdomys* and *Mus* and extracted isolated response features from the peristimulus time histograms (PSTHs) of light-responsive neurons using a sparse principal component analysis (sPCA; Figure S2; see STAR Methods). We then clustered the data by applying a Gaussian mixture model to the feature set and optimized the cluster number by minimizing the Bayesian information criteria (Figure S2B). This approach produced 15 functional clusters, which varied in their response sign, temporal frequency tuning, and/or contrast sensitivity function (Figures 1B, 1C, S3, and S4). Mapping the location of all recorded units across the dLGN ruled out any systematic sampling bias across clusters (Figure S1C).

As expected from earlier work,^16–18^ we found response types corresponding to transient ON (clusters 4, 5, 9, and 13), transient OFF (10 and 11), sustained ON (12, 14, and 15), and ON-OFF responses (1, 2, 3, 7, and 8). Within those categories, there was further diversity in temporal and contrast sensitivities. Clusters 1, 3, 5, 9, and 13 showed band-pass temporal frequency tuning, with the remainder showing low-pass tuning. Clusters 5, 13, and 14 showed the highest contrast sensitivity (sensitivity defined by half-maximum of Naka-Rushton curve, see STAR Methods), whereas 4 and 6 had the lowest sensitivity, and clusters 2 and 5 showed saturating contrast sensitivity curves. Suppressed-by-contrast responses were also identified (clusters 6 and 8).

We next compared the proportion of neurons from *Rhabdomys* or *Mus* that were assigned to each functional cluster (Figure 1D). Most clusters (12/15) contained units from both *Mus* and *Rhabdomys*, and, where this was the case, the mean response profile remained consistent between species (Figure S2H). However, three types appeared to be unique to one species (1, 3, and 15) and frequencies differed by >5-fold for others (2, 5, 9, 12, 13, and 14; Figure 1D). Accordingly, the overall frequency distribution of units across clusters was highly significantly different between species (chi-squared test, *p* < 0.001). This pattern was consistent across experimental animals and robust to controls for cluster number, batch effects, and critical analysis parameters (Figures S2C–S2G).

The 3 most-common clusters in *Rhabdomys* (transient ON-OFF; clusters 1–3) were represented by only 4 units in *Mus*. Conversely the clusters with highest *Mus* representation (sustained ON; 12–15) were sparsely represented in *Rhabdomys* (21 units across these 4 clusters). To independently verify these prominent differences between the two species, we separately classified dLGN response units according to the kinetics and polarity of their response to a 2-s step (2-s 97% contrast step every 20 s) (STAR Methods; Figures 1E, 1F, and S3). The distribution of units across the resultant categories was again significantly different between species (chi-squared test *p* < 0.001), confirming that the most-common response type was ON-OFF in *Rhabdomys* and sustained ON in *Mus*.

Among the responses to the chirp element of the stimulus >50% of *Rhabdomys* units but only ~33% of *Mus* units were from clusters displaying band-pass temporal frequency tuning (1, 3, 5, 9, and 13). Accordingly, calculations across the whole dLGN population confirmed a preference for higher frequencies in *Rhabdomys* (Figure S4). There was a slight but significant shift toward increased contrast sensitivity in *Rhabdomys* compared with *Mus* (Kolmogorov-Smirnov *p* < 0.01; Figures S4C, S4D, S4G, and S4H). *Mus* neurons were over-represented in clusters with the lowest contrast sensitivity (5, 13, and 14), whereas *Rhabdomys* neurons predominated clusters with high contrast sensitivity (4 and 6). Clusters showing saturating responses (2 and 5) were also predominant in *Rhabdomys*. Suppressed-by-contrast profiles were detected in both species, albeit at low frequency (clusters 6 and 8).

### Enhanced spatiotemporal resolution in *Rhabdomys* dLGN

Using a behavioral assay of spatial vision,^19^ we found that *Rhabdomys* responded to sinusoidal gratings at frequencies >2-fold higher than the established acuity of *Mus*^20^ (Figure S5). To relate this result to acuity as defined electrophysiologically, we first mapped receptive fields (RFs) using a binary dense noise stimulus (5 Hz). Using spike-triggered averages (STAs), we were able to reconstruct RFs in 41% and 64% of light-responsive neurons in *Mus* and *Rhabdomys*, respectively. Spatial RFs had robust center responses, which were delineated as ON or OFF polarity (Figures 1G–1I and S6). Robust opposing surround responses were rare in both species, consistent with previous work in *Mus*,^21^ and are not further quantified here. In *Mus*, the mean RF diameter was consistent with prior estimates (median: 5.54^16,17^; Figure 1I). In *Rhabdomys*, this value was only 6% smaller (median: 5.20; Kolmogorov-Smirnov *p* = 0.036) and the distribution of RF sizes in each species covered a similar range. The *Rhabdomys* dLGN displayed higher temporal fidelity, with the temporal filter in the RF center having significantly shorter latency in this species (median = 134 and 186 ms in *Rhabdomys* and *Mus*, respectively; Kolmogorov-Smirnov test: *p* < 0.001; Figure 1J). Although individual RFs were similar in the two species, there was a greater overlap of RFs in *Rhabdomys* dLGN neurons. This is consistent with the enhanced dLGN volume and possibly indicates species differences in convergence/divergence from RGCs to dLGN (Figure 1K).

**Figure 2:**
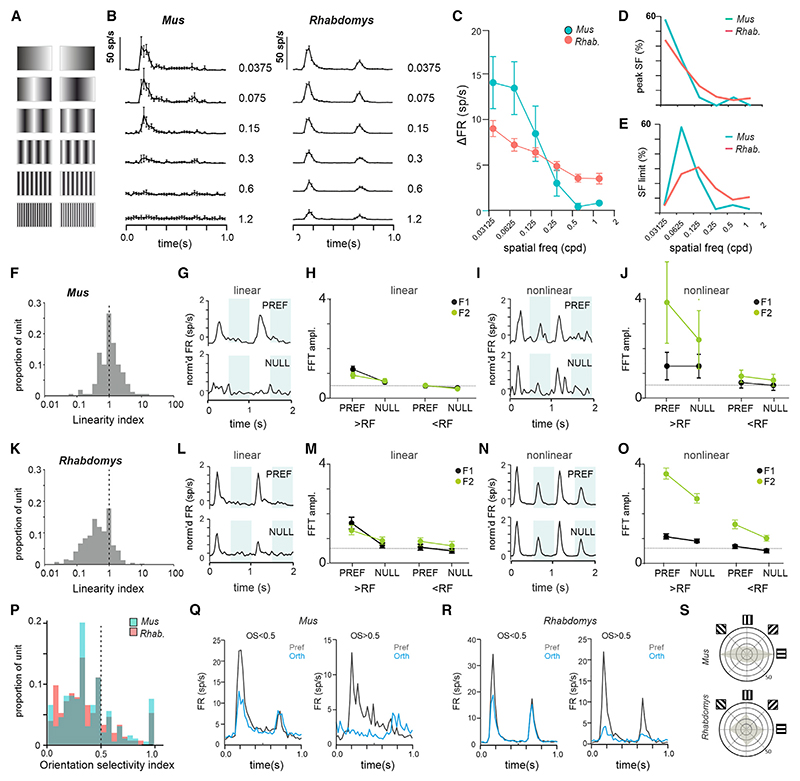
Spatial-frequency tuning and response linearity in *Mus* and *Rhabdomys* (A) Schematic of inverting grating stimuli of increasing spatial frequency. (B) Mean ± SEM PSTH for 1 Hz inverting grating stimuli at 6 spatial frequencies (0.0375, 0.075, 0.15, 0.3, 0.6, and 1.2 cpd) for *Mus* (left) and *Rhabdomys* (right). (C) Amplitude of response to increasing frequency-inverting gratings at preferred orientation/phase, shown for *Mus* (cyan) and *Rhabdomys* (red); data show mean ± SEM. (D and E) Distribution of % of neurons with peak response at a particular frequency (D), and with a threshold to response at a particular spatial frequency (E), shown for *Mus* (cyan) and *Rhabdomys* (red). (F) Distribution of linearity index (LI) in *Mus*. (G) Mean normalized response of linear neurons in preferred (top) or null (lower) phase, in response to 2 grating inversions. Shaded regions indicate inversions. (H) fft amplitude in preferred and null phase of the response, for spatial frequencies greater than (left) or less than (right) the receptive field size and for F1 (black) and F2 (green) frequency components. (I and J) As in (G) and (H) but for non-linear responses in *Mus*. (K–O) As in (F)–(J) but for *Rhabdomys*. (P) Distribution of orientation selectivity index (OSI) for *Mus*. (Q and R) Mean response of *Mus* (Q) and *Rhabdomys* (R) neurons with OSI < 0.5 (left) and OSI > 0.5 (right) in response to inverting grating at optimal spatial frequency, in preferred (black) and null (red) phase. (S) Distribution of preferred orientation of bars for OS neurons (note double plot from 180° to 360°) for *Mus* and *Rhabdomys* (top and bottom, respectively). See also Figures S5 and S7 and Video S1.

We then compared spatial acuity for neurons in *Mus* and *Rhabdomys* dLGN by presenting inverting gratings at 6 spatial frequencies (each at two phases and at 4 orientations; 1 Hz; Michelson contrast = 97%). Quantifying the change in firing rate as a function of spatial frequency at each neuron’s optimal phase/orientation (see STAR Methods) revealed a significant increase in the sensitivity of *Rhabdomys* dLGN neurons to higher spatial frequencies compared with *Mus*. Thus, at their preferred phase/orientation, most *Mus* neurons showed low-pass tuning across the range of spatial frequencies tested, responding up to around 0.6 cycles/degree (Figures 2A, 2C, and 2D), whereas most *Rhabdomys* neurons remained responsive to inverting gratings at the highest spatial frequency tested (1.2 cycles per degree [cpd]; Figures 2B–2E), much smaller than the predicted RF size (~3°–8°).

The higher frequency of ON-OFF units in *Rhabdomys* suggests that enhanced non-linear spatiotemporal integration could contribute to its higher acuity.^22^ To further examine this, we quantified the response amplitude to inverting gratings that were larger than the calculated RF size of each neuron (identifying the optimal phase and stimulus orientation). We then calculated a linearity index (LI), a ratio of the response at the fundamental frequency of the stimulus (F1, 1 Hz), to the second harmonic (F2; 2 Hz) in response to this stimulus in each species (Figures 2F–2O). Around 25% of neurons were “non-linear” in *Mus* dLGN (similar to Piscopo at al.^17^), in contrast with at least 60% in *Rhabdomys*. In both species, non-linear units responded to inverting gratings at spatial frequencies that were smaller than their RF sizes (Figures 2I, 2J, 2N, and 2O), whereas linear units did not (Figures 2G, 2H, 2L, and 2M).

### Orientation/direction selectivity and responses to motion

Analysis of the inverting grating responses enabled us to determine the degree of orientation selectivity by calculating an orientation selectivity index (OSI; STAR Methods) for each neuron at its preferred spatial frequency and phase. The range of OSI values in the *Mus* dLGN was consistent with previous reports,^16,17^ and a similar distribution was found in *Rhabdomys* (Figures 2P–2R). At a conservative threshold of OSI = 0.5, 10% and 11% of light-responsive neurons in *Mus* and *Rhabdomys*, respectively, were classed as orientation selective. In OSI neurons, we found a strong preference for horizontally orientated bars in *Mus*, whereas *Rhabdomys* showed a more even distribution around the tested orientations (Figure 2C); however, the low numbers of OS neurons make it hard to attribute significance to those differences.

We applied a moving bar stimulus to explore motion sensitivity and direction selectivity. ~90% of light-responsive neurons in each species responded to this stimulus with a phasic change in firing. Responsive neurons showed changes in firing at the leading and/or trailing edge of the bar when moving over its RF (see representative neurons in Figures S7A and S7B). Consistent with our evidence of enhanced non-linear spatiotemporal summation in *Rhabdomys*, there was a bias toward responses to both the leading and trailing edge in this species. This could be seen most clearly in the spike-triggered average within an individual neuron’s RF (Figures S7C and S7D). We calculated a direction selectivity index (DSI) for neurons that responded to the moving bar. In a subset of neurons, the strength of responses was dependent on the direction of movement (Figure S7B), consistent with previous reports in *Mus* dLGN^16,17^; however, there was a similar distribution of DSIs in both species (Figure S7E). We did not find any statistical difference in the preferred direction of motion between species (Figure S7F; chi-squared test *p* < 0.001), though *Rhabdomys* showed a bias toward motion sensitivity in the dorso-temporal and ventro-nasal directions.

### ERG recordings in *Mus* and *Rhabdomys*

**Figure 3:**
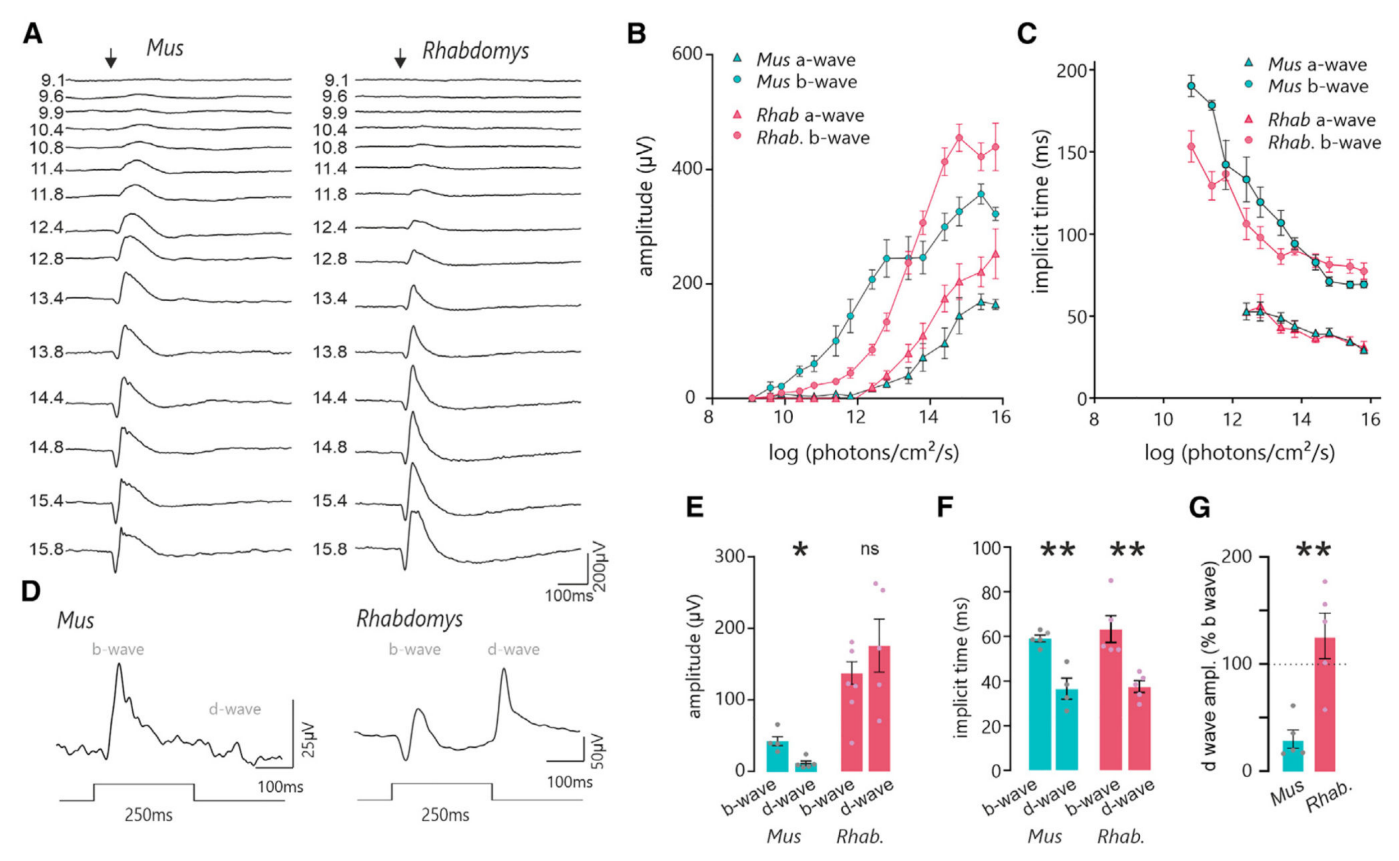
Retinal sensitivity to light in *Mus* and *Rhabdomys* (A) Representative traces of dark-adapted flash electroretinograms in *Mus* and *Rhabdomys* (left and right, respectively). Flash intensities range from 9.1 to 15.8 log photons/cm^2^/s (shown to left of ERG traces). Arrow indicates flash onset. (B and C) Amplitude (B) and implicit time (C) of a and b waves recorded in *Mus* (cyan) and *Rhabdomys* (red). Data shown mean ± SEM, *n* = 5 animals per species. (D) Representative ERG traces recorded from *Mus* and *Rhabdomys* in response to 250 ms light step (time course indicated below; light intensity 14 log photons/ cm^2^/s). (E) Quantification of b-wave (light onset) and d-wave amplitudes (light offset) in response to 250 ms light step. Data compared with repeated measures two-way ANOVA finding significant effects of species and b-/d-wave:species interaction. *p < 0.05 for post hoc Bonferroni test. Data show mean ± SEM, *n* = 5. (F) Quantification of b-wave and d-wave implicit time in response to 250 ms light step. Data compared with repeated measures two-way ANOVA finding significant effects of b- vs. d-wave implicit time. **p < 0.01 for post hoc Bonferroni test. Data show mean ± SEM, *n* = 5. (G) d-wave amplitude expressed as % of b-wave amplitude. Data compared with unpaired t test. **p < 0.01. For (E)–(G): *Mus* (cyan) and *Rhabdomys* (red). Data show mean ± SEM, *n* = 5.

Although the cortical visual code is sculpted in the dLGN,^18^ its fundamental properties are inherited from the retina. Given the marked increase in appearance of OFF excitation in *Rhabdomys* dLGN, we therefore turned to *in vivo* electroretinography to determine whether this was also apparent in the retinal light response. Three components of the electroretinogram (ERG) are relevant: the a-wave is derived from photoreceptors (PRs), the b-wave from ON bipolars, and the d-wave from OFF bipolars.^23^ In response to brief flashes of light under dark-adapted conditions, the a-wave was larger in *Rhabdomys* at high intensities and higher in *Mus* at low intensities, consistent with the relative paucity of rods, which are more sensitive than cones, in *Rhabdomys*^10,11^ (Figures 3A–3C). Similarly, the b-wave implicit time (latency to peak), which reflects ON bipolar cell (BC) activity, was reduced in *Rhabdomys*, which would be consistent with a reduced contribution of the more sluggish rod pathway to ON responses. Turning to the question of OFF excitation, we recorded ERGs in response to an extended (250 ms) step under light-adapted (cone isolating) conditions. This stimulus can reveal the activity of ON and OFF cone BCs as separate b- and d-waves associated with the appearance and disappearance of the light step, respectively. In accordance with the literature,^23^ we found that the light pulse ERG is dominated by the b-wave in *Mus* (Figures 3E–3G). Conversely, in *Rhabdomys*, d-waves were at least as prominent (Figures 3E–3G), consistent with a strong contribution of OFF BCs and a realignment toward OFF excitation from the earliest step of visual signal transduction in this species.

### Comparison of *Mus* and *Rhabdomys* retinal cell classes and types

**Figure 4:**
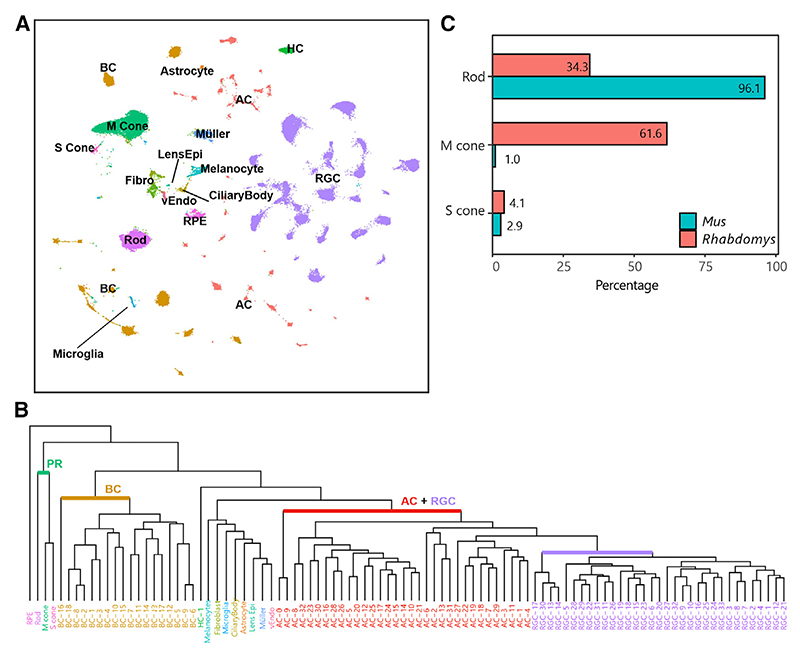
A retinal cell atlas for *Rhabdomys* (A) Transcriptionally distinct clusters of *Rhabdomys* retinal cells visualized using uniform manifold approximation and projection (UMAP).^29^ Cells are colored by class identity (RPE, retinal pigment epithelial cell; vEndo, vascular endothelial cells; Lens Epi, lens epithelial cells; Fibro, fibroblasts). (B) Dendrogram showing transcriptional relationships of *Rhabdomys* cell clusters, with major clades corresponding to cell classes. (C) Bar chart indicating proportion of photoreceptor types in *Rhabdomys* (red) and *Mus* (cyan).

To understand the distinct coding properties in the primary visual pathway of *Rhabdomys* and *Mus*, we compared retinal neuronal types of the two species, using atlases derived from single-cell RNA sequencing (scRNA-seq) and single-nucleus RNA-seq.^24–27^ The *Rhabdomys* atlas^28^ (Figure 4) contained 65,930 nuclei from 2 *Rhabdomys*, which could be classified via standard computational procedures into the five retinal neuronal classes (PRs, horizontal cells [HCs], BCs, amacrine cells [ACs], and RGCs) as well as multiple glial types (Müller glia, astrocytes, and microglia) and endothelial and retinal pigment epithelial cells (Figure 4A). Each neuronal class could be further divided into transcriptomically distinct clusters: 3 PR, 1 HC, 18 BC, 33 AC, and 33 RGC clusters (Figure 4B). Altogether, the atlas included over 100 retinal clusters, representing putative cell types. Within each class, nearly all *Rhabdomys* clusters mapped in a specific fashion to *Mus* types (see below). This correspondence allowed us to transfer cell-type labels of the better-studied *Mus* to *Rhabdomys*, facilitating comparison between the two species.

### *Rhabdomys* retina is cone dominated

Analysis of PR transcriptomes confirmed and quantified the known shift in rod:cone ratio between *Mus* and *Rhabdomys*: ~3% of *Mus* PRs but >65% *Rhabdomys* PRs are cones, a >20-fold difference (Figure 4C). Also anticipated was the degree of cone opsin co-expression. ~40% of *Mus* medium-wavelength-sensitive (MWS) cones express short-wavelength-sensitive (SWS) opsin at low levels,^30^ whereas this was true for only ~1.3% of *Rhabdomys* MWS-cones (112 out of 8,622 cones), with the majority (92.7%) expressing only MWS opsin.

### A reduction in rod BCs and increased proportion of OFF BCs in *Rhabdomys* inner retina

**Figure 5:**
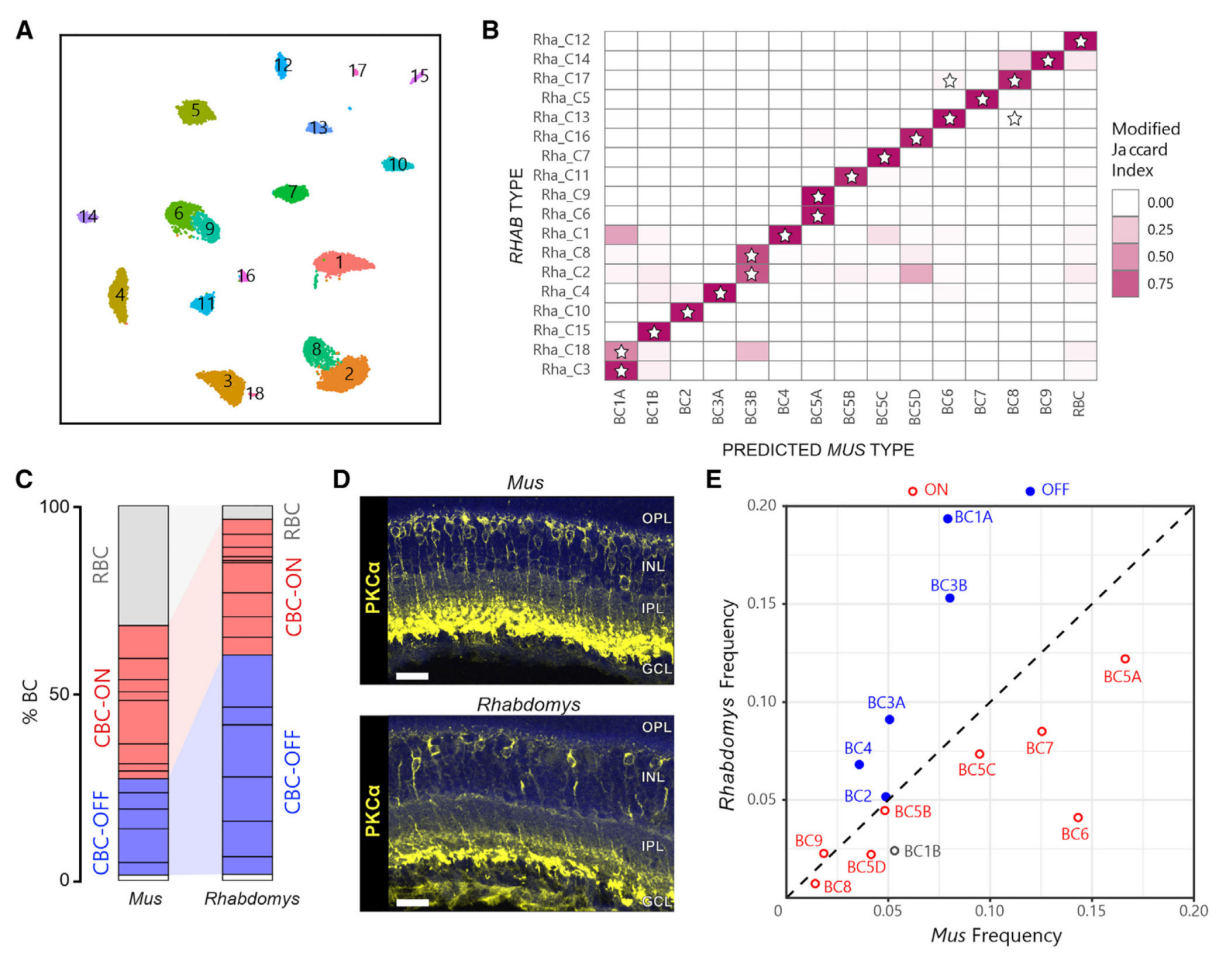
Proportions of BC types in *Rhabdomys* and *Mus* (A) *Rhabdomys* BCs clustered separately and displayed using UMAP. Cells colored by cluster identity. (B) Confusion matrix indicating the transcriptional correspondence between *Rhabdomys* BC cluster identity (rows) and classifier-assigned *Mus* BC type identity (columns). Cells are colored based on the modified Jaccard index (color bar, right), which ranges from 0 (no correspondence) to 1 (perfect correspondence; see STAR Methods for details). Pairs of *Rhabdomys* clusters and *Mus* types that belong to the same BC “orthotype” in Hahn et al.^28^ are indicated by a star. The preponderance of stars along the diagonal indicates a high concordance between the correspondence analysis presented here and the orthotype analysis in Hahn et al.^28^ (C) Bar graph showing percentage of BCs assigned rod BC (gray), cone ON BC (red), cone OFF BC (blue), or BC1B (white) cell types in *Mus* and *Rhabdomys*. Dark horizontal lines within each subclass demarcate distinct types. Percentages for biological replicates are shown in Table S1. (D) Transverse sections of *Mus* and *Rhabdomys* stained with antibodies to PKCα. INL, inner nuclear layer; IPL, inner plexiform layer; GCL, ganglion cell layer. RBC axon terminals are labeled intensely. Scale bar, 20 μm. (E) Scatterplot comparing the relative frequency of ON (red) and OFF (blue) cone BC types (labeled according to *Mus*) between the two species. BC1B, which cannot be classified as either ON or OFF, is labeled gray. See also Figure S8 and Tables S1 and S2.

BCs can be subdivided into those that depolarize (ON) or hyperpolarize (OFF) to illumination. Cones innervate both ON and OFF BCs, whereas all rod BCs are ON BCs. Clustering the ~11,400 *Rhabdomys* BC transcriptomes generated 18 putative types (Figure 5A). Based on known markers (Figure S8A),^24,31^ these types comprised 7 OFF cone BC types (expressing FRIK1 and TRPM3^24,31^), 9 ON cone BC types (expressing GRM6 and ISL1^24,31^), and 1 rod BC type. The final type, BC1B, receives no direct PR input^24,32^ so cannot be confidently classified as either ON or OFF. A supervised classification analysis based on transcriptomic signatures indicated a predominantly 1:1 correspondence between the *Rhabdomys* BC types and *Mus* BC types (Figure 5B). The patterns of correspondence were consistent with the results of our recent comparative analysis of BC types across 13 mammals using an integrative clustering approach.^28^

Three notable differences in BC composition between species were evident. First, the 18 BC types in *Rhabdomys* exceeded the 15 that have been identified and validated in *Mus. Mus* types BC1A, 3B, and 5A each mapped to two *Rhabdomys* types (Figure 5B). Members of each pair (C3 and C18 for BC1B, C2 and C8 for BC3B, and C6 and C9 for BC5A) differentially expressed multiple genes (Figure S8B), supporting their identity as distinct types. Second, rod BCs comprised around 3.5% of the total BCs in *Rhabdomys*, in contrast to ~43% in *Mus*, correlating with the difference in rod:cone ratio between species (Figure 5C; Table S1). Immunostaining with the rod BC (RBC) marker Protein kinase C alpha (PKCα) validated this difference (Figure 5D), with approximately 18× as many RBCs in *Mus* vs. *Rhabdomys* (7,771 ± 1,111 vs. 413 ± 55 cells/mm^2^). Third, among cone BCs (that is, separate from the difference in rod BC frequency), there are more OFF BCs and fewer ON BCs in *Rhabdomys* than in *Mus*. The four most-abundant cone BC types in *Rhabdomys* retina (C1–4; clusters are numbered in order of their abundance) all corresponded to OFF types in *Mus*. Conversely, all but one of the ON cone BC types were more abundant in *Mus* (Figure 5E; Table S2). This redistribution of CBC types would predict an enhanced OFF-type response at the first retinal synapse, consistent with the enhanced photopic d-wave observed in the ERG (Figure 3).

### Increased abundance of OFF and ON-OFF RGCs in *Rhabdomys*

**Figure 6:**
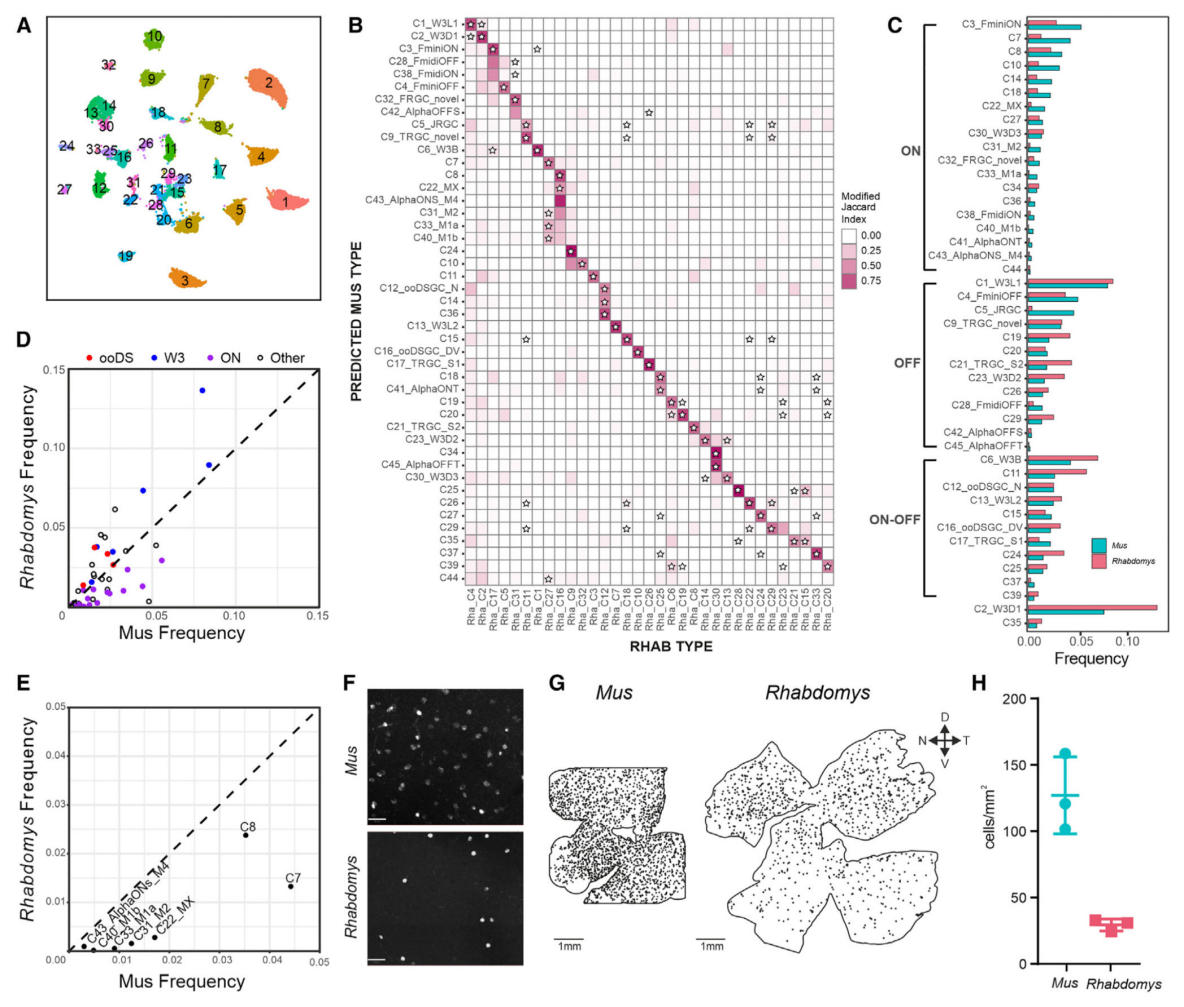
Proportions of RGC types in *Rhabdomys* and *Mus* (A) *Rhabdomys* RGCs were clustered separately and displayed using UMAP. Cells are colored by cluster identity. (B) Confusion matrix indicating the transcriptional correspondence between *Rhabdomys* RGC cluster identity (columns) and *Mus* RGC type identity (rows). Cells are colored based on the modified Jaccard index (color bar, right), as in (B). Stars indicate pairs of *Rhabdomys* clusters and *Mus* types that belong to the same orthotype in Hahn et al.^28^ This figure shows results of a classifier trained on *Rhabdomys* data. Correspondence was similar when the classifier was trained on *Mus* data. (C) Bar graph showing the relative frequency of each *Mus* RGC type in both the species. Types are grouped by response polarity—ON, OFF, or ON-OFF—based on results in Tran et al.,^25^ Goetz et al.,^33^ Rousso et al.,^34^ and Huang et al.^35^ Percentages for biological replicates are shown in Table S3. (D) Scatterplot comparing relative frequency of RGC types between *Rhabdomys* and *Mus*. Response polarity is as shown in (C). All 6 types of the W3 subclass^25,36^ are more abundant in *Rhabdomys* than *Mus*. (E) Scatterplot showing the reduced frequency of putative ipRGC types in *Rhabdomys* (y axis) compared with *Mus* (x axis). (F) Representative HCR-FISH of *Mus* and *Rhabdomys* retinal wholemounts, with probe for *Opn4* (species specific). Scale bar, 50 μm. (G) As in (B) but showing location of *Opn4*+ RGCs across whole retina of *Mus* and *Rhabdomys*. Scale bar, 1 mm. Orientation shown to right. (H) Number of *Opn4*+ cells/mm^2^ in *Mus* and *Rhabdomys* retinal wholemounts (*n* = 3; lines show mean ± SEM). Data compared with unpaired t test; **p < 0.01. See also Figure S9 and Table S3.

Re-clustering the 26,500 *Rhabdomys* RGCs transcriptomes yielded 33 clusters (Figure 6A), which is substantially lower than the 45 molecularly distinct RGC types in *Mus*. However, supervised classifiers trained on either *Mus* or *Rhabdomys* data indicated that all known 45 *Mus* RGC types were represented among the *Rhabdomys* clusters (Figure 6B). Thus, in several instances, a *Rhabdomys* cluster was composed of a group of closely related *Mus* types. The difference in resolution was likely due to differences in cell number (26,539 in *Rhabdomys* vs. 35,699 in *Mus*), sequencing depth, and/or modality (single cell vs. single nucleus).

RGCs are conventionally classified as ON, OFF, or ON-OFF types depending on whether they are excited by increases or decreases in light intensity or both. Previous studies have combined morphological, physiological, and transcriptomic data to characterize *Mus* RGCs.^25,33–35^ Based on those results, we were able to assign 43 *Mus* types to one of these three categories (Figure 6C). Lacking physiological data from *Rhabdomys* RGCs, we used our supervised classification model to provisionally categorize *Rhabdomys* RGCs as ON, OFF, or ON-OFF, based on their assigned *Mus* label. The 8 most-abundant putative ON types in *Mus* were all under-represented in *Rhabdomys*, and most OFF or ON-OFF types were more abundant in *Rhabdomys* than in *Mus*, including all four of the known ON-OFF direction-selective types (ooDSGCs; Figures 6C and 6D; Table S3). This bias is consistent with the preponderance of OFF BCs in retina and OFF and ON-OFF responses in dLGN noted above. Interestingly, orthologs of a subclass of *Mus* RGCs defined by expression in the “THWY3” transgenic line^25,36,37^ were enriched in *Rhabdomys* (Figure 6D); 2 of these types are OFF, 2 ON, 1 ON-OFF, and 1 unknown.

### Depletion of ipRGCs in *Rhabdomys*

Several types of melanopsin (*Opn4*) expressing intrinsically photosensitive RGCs (ipRGCs) have been characterized in *Mus*.^38–41^ Our *Mus* RGC atlas identifies 5 ipRGC types, which we have provisionally called M1a, M1b, MX, M2, and M4. Two additional *Mus* RGC types, C7 and C8, are closely related to the ipRGCs. Both express the subclass-defining transcription factor *Eomes*, plus *Opn4* at low levels.^25^ C7 has recently been provisionally identified as the M6 type.^42^ *Mus* ipRGC types co-mapped to *Rhabdomys* clusters C16 and C27 (Figure 6B). However, for all these types, the relative abundance was substantially lower in *Rhabdomys* than in *Mus* (Figure 6E), which is the likely reason that they did not form separate clusters.

To validate these results, we used fluorescence *in situ* hybridization chain reaction (HCR-FISH) to compare *Opn4+* RGCs in retinal wholemounts from the two species (Figure 6F). Using an automated cell-detection method (Figure S9), we found that the number of *Opn4+* cells per unit area was substantially lower in *Rhabdomys* than *Mus* (Figures 6G and 6H), indicating that both the absolute density of ipRGCs (5× lower) and their representation as a function of the total RGC population were greatly reduced in *Rhabdomys* compared with *Mus. Opn4+* RGCs shared expression of the transcription factor, *Eomes*, which is expressed in all ipRGC types^43^ (Figure S9D). It remains to be determined whether all ipRGC types are equally depleted, though, intriguingly, *Opn4+* cells in *Rhabdomys* seem to be biased toward small soma sizes and high Opn4 expression (Figures S9E–S9G), which could signal that certain subtypes are especially impacted (dLGN-projecting M4 types have large somas and weak Opn4 expression in mice^40,44^).

## Discussion

We combined physiological and transcriptomic techniques to understand how the visual systems of closely related murid rodents are adapted to divergent visual ecology. Day-active *Rhabdomys* have thicker inner retinas and larger dLGNs than nocturnal *Mus*. Our data reveal that although they apply this extra capacity to provide denser coverage of the visual scene, this does not amount to simply “more of the same” visual code seen in *Mus*. Rather, we find a marked realignment toward non-linear spatiotemporal summation in the *Rhabdomys* dLGN. This is apparent in a shift from predominantly ON responses in *Mus* toward biphasic, ON-OFF responses and increased abundance of units able to track gratings sufficiently fine to fall entirely within their RF center in *Rhabdomys*. These inter-species differences in visual code and acuity are accompanied by selective expansions in the abundance of OFF BCs and both OFF and ON-OFF RGCs in *Rhabdomys* compared with *Mus*.

In principle, the increased capacity of the *Rhabdomys* early visual system could have been employed in numerous ways. One option would have been to transmit a higher-fidelity, less-filtered representation of the scene to the cortex. That would facilitate richer computations and interpretation at higher levels and is the strategy often employed by other species with enhanced visual systems such as primates, where a relatively unprocessed visual code is coupled with an expansion of the visual cortex.^45–47^ However, we find that such simple “pixel encoders” (characterized by sustained responses and linear spatial summation) are substantially less common in *Rhabdomys* than *Mus* (Figures 2 and S3). At the other extreme, *Rhabdomys*’ extra capacity could plausibly be applied to allow more diverse computations in the early visual system, parsing the visual scene in more complex ways.^48,49^ Our cell atlas analysis provides some support for that proposition, as there is evidence of increased diversity in the BC population with *Mus* subtypes BC1A, 3B, and 5A, each represented by two transcriptionally distinct clusters in *Rhabdomys*. However, we did not observe a corresponding increase in diversity in the *Rhabdomys* visual code. Thus, unbiased clustering returned around 14 functionally distinct channels in the dLGN of each species (with the proviso that more complexity is expected with a wider array of visual stimuli and/or in distinct RGC targets).

Given the alternatives, what advantage might *Rhabdomys* attain by selectively increasing representation of units with non-linear spatiotemporal summation, especially as such non-linear feature detectors are often more abundant in the retinas of animals that invest less neural resources in (form) vision (e.g., Zhang et al.^37^). One possibility is that it represents an efficient approach to enhance spatiotemporal resolution. We find that RF sizes are only marginally finer in *Rhabdomys* compared with *Mus*. With this constraint in mind, natural selection can take two routes to exploit the additional information of *Rhabdomys* to increase spatiotemporal acuity. On the one hand, it can increase the degree of overlap in RF between units to achieve denser coverage of the scene at neuronal population level. We find that this is indeed a feature of the *Rhabdomys* visual code. Alternatively, it can employ non-linear spatiotemporal summation to allow individual neurons to respond to patterns within their RF, achieving greater acuity at the single-unit level. *Rhabdomys* appear to exploit this opportunity too, as we find that realignment of the visual code toward non-linear summation allows many units to respond to high-frequency gratings.

The primary features of the thalamic visual code are inherited from the retina, and species differences in the visual code that we observe are accompanied by some substantial differences in retinal cellular composition. Most obviously, the *Rhabdomys* retina has many more cone PRs, and this is achieved by selective expansion of the M-cone opsin-expressing cones (the fraction of PRs expressing S-cone opsin hardly differs between species). The shift toward cones is expected for a more day-active species. That this is restricted to M-cone opsin-expressing cones is perhaps associated with *Rhabdomys*’ combination of UV-sensitive S-cone opsin with a lens that filters out UV-wavelength light.^11^

Second-order neurons are also substantially different in *Rhabdomys*, with no overlap in the 3 most-abundant BC types in each species. The switch to a cone-rich retina in *Rhabdomys* is accompanied by a large decrease in the relative proportion of rod BCs. This may be predicted, given the cone bias of the *Rhabdomys* retina, but recent analysis reveals that rod BCs can be numerous, even in species with high cone density.^28^ Evidence that cones can signal via rod BCs supports the contention that rod BCs do not necessarily become redundant when rod numbers fall. Nevertheless, both anatomical and physiological characteristics of cone BCs facilitate information transfer at higher spatiotemporal resolution.

The decrease in rod BC frequency in *Rhabdomys* compared with *Mus* is accompanied by an increase in cone BC frequency. However, the increase is not distributed equally across cone BC types. Instead, it appears disproportionately in OFF BCs (in order of fractional abundance BC3B, BC4, BC1A, and BC3), whereas cone ON BCs occupy equivalent fractions of the BC population in the two species. The enrichment of OFF BCs in *Rhabdomys* is consistent with the enhanced ERG d-wave amplitude (measuring OFF responses) and the preponderance of OFF excitation responses in the dLGN of this species. A plausible function of the increase in OFF BC frequency facilitates non-linear spatiotemporal summation and, indeed, the two most-expanded BC classes in *Rhabdomys* (BC3B and BC4) have characteristics well suited to this purposes (small dendritic and axonal fields and transient responses^50,51^). But realignment toward OFF BCs may bring additional benefits. OFF BCs respond faster than ON BCs, allowing the possibility not only for faster visual reflexes but also higher acuity.^52^ As a day-active species, *Rhabdomys* may place a higher premium on detecting shadows (attributed to OFF pathways^53^) and, in a more general sense, the negative spatial contrast to which natural scenes are biased^54^ than high-sensitivity vision (attributed to ON pathways^55^). Finally, the enhanced capacity of OFF pathways could support better ability to detect overhead (dark) looming stimuli indicative of aerial predators.^56^

Species differences in the RGC population are more nuanced than those in PR or BC makeup but are in accordance with the changes in visual code that we observe. Thus, in the *Rhabdomys* atlas, RGC types characterized by ON responses in mice are relatively under-represented in favor of those with OFF and ON-OFF responses. We also find a significant reduction in the population of melanopsin-expressing ipRGCs compared with *Mus*. The significance of this realignment remains unclear, and how specific subtypes are impacted by this reduction remains an open question. It is interesting to note that ipRGC numbers are reduced in other day-active species (including humans)^57–59^ but notably over-represented in the naked mole-rat^60^ (~90% RGCs) and nocturnal microbat (~15%),^61^ indicating a possible link between ecological niche and ipRGC cell complement.

The *Mus* vs. *Rhabdomys* comparison represents a case study in how sensory systems can adapt to different ecology. Despite their similarity in phylogeny, size, and diet, these species have evolved markedly different visual systems. *Rhabdomys* is adapted to exploit the higher signal:noise of visual signals in the day, with its cone-rich retina and expansion in the thickness of the inner retina and dLGN. We find that it applies this additional information capacity in both predictable and more surprising ways. Thus, in addition to simply achieving greater density of coverage of the visual scene, changes in composition of the retinal cell population drive a realignment in favor of OFF responses and non-linear spatiotemporal summation, which increases spatial resolution in the *Rhabdomys* visual system and may provide other benefits. Our data thus show how evolutionarily advantageous changes in computational outcome do not require invention of new types but can be produced by selective expansion/ contraction of common cell types comprising a neural circuit.

## Resource Availability

### Lead contact

Further information and requests for resources and reagents should be directed to and will be fulfilled by the lead contact, Annette Allen (annette.allen@manchester.ac.uk).

### Materials availability

The study did not generate new unique reagents.

### Data and code availability

- The raw and processed sequencing data produced in this work have been deposited at Gene Expression Omnibus (GEO) under accession number GEO: GSE237210 and are publicly available as of the date of publication. Electrophysiological data reported in this paper will be shared by the lead contact upon request.
- All original code has been deposited at Zenodo (https://zenodo.org/record/8067826) and on GitHub (https://github.com/shekharlab/RetinaEvolution) and is publicly available as of the date of publication.
- Any additional information required to reanalyze the data reported in this paper is available from the lead contact upon request.

## Star★Methods

### Key Resources Table

**Table:** 

REAGENT or RESOURCE	SOURCE	IDENTIFIER
Antibodies		
primary antibody rabbit anti-melanopsin antibody	Advance Targeting Systems	UF006; RRID:AB_1608077
primary antibody rabbit anti-PKCα	Abcam	ab32376; RRID:AB_777294
Deposited data		
Raw and processed sequencing data	Gene Expression Omnibus	GEO: GSE237210; GEO: GSE137400; GEO: GSE81905
Experimental models: Organisms/strains		
*Mus musculus*	University of Manchester Biological Services Facility inbred strain, obtained from Envigo.	C57BL/6J; RRID:IMSR_JAX:000664
*Rhabdomys pumilio*	University of Manchester Biological Services Facility	F10 descendants of wild-derived striped mice (*R. pumilio*, originating from Goegap Nature Reserve, South Africa, S 29**°** 41.56′, E 18**°** 1.60′) were obtained from a captive colony at the University of Zurich (Switzerland) and are maintained at Manchester University.
Software and algorithms		
Python	N/A	https://www.python.org/
QuPath	N/A	https://qupath.github.io/
MATLAB	The Mathworks	https://www.mathworks.com/products/matlab.html
Signal	CED	https://ced.co.uk/products/signal
Psychopy	N/A	https://www.psychopy.org/ ^62^
Arduino	N/A	https://www.arduino.cc/
R	N/A	https://www.r-project.org/
FlyCapture2 SDK	N/A	https://www.flir.com
Offline Sorter	Plexon	https://plexon.com/products/offline-sorter/
SmartBox recording system	Neuronexus Technologies, Inc.	https://www.neuronexus.com/products/smartbox-pro/
CircStat, MATLAB toolbox	N/A	Berens^63^
SPaSM, MATLAB toolbox	N/A	Sjöstrand et al.^64^
Kilosort v3 (in MATLAB)	N/A	Pachitariu et al.^65^
Seurat v4.3.0	Satija laboratory	https://satijalab.org/seurat/; Stuart et al.^66^
Xgboost (R package)	N/A	Chen and Guestrin^67^
Analysis scripts for snRNA-seq data	Shekhar laboratory	https://zenodo.org/record/8067826 and https://github.com/shekharlab/RetinaEvolution/
Other		
Probes for HCR™ RNA-FISH	Molecular Instruments	https://Molecularinstruments.com/

### Experimental Model and Study Participant Details

#### Animals

Animal care was in accordance with the UK Animals, Scientific Procedures Act of 1986, and the study was approved by the University of Manchester ethics committee. Animals were housed on a 12h:12h light:dark cycle at 22°C with food and water available ad libitum. All experiments were performed in adult *Rhabdomys pumilio* or C57BL6J mice (aged 3–8 months; mixed male/female cohort).

### Method Details

#### *In vivo* electrophysiology

*In vivo* electrophysiological recordings were performed in 6 *Rhabdomys* and 5 *Mus* (male), using methods described previously.^11^ Recordings were made roughly between ~3 and 9 hours after light onset. Anaesthesia was induced with 2% isofluorane in oxygen, and maintained with an intraperitoneal injection of urethane (1.6 g kg – 1, 30% w/v; Sigma-Aldrich). A topical mydriatic (tropicamide 1%; Bausch and Lomb) and mineral oil (Sigma-Aldrich) were applied to the left eye prior to recording. After placement into a stereotaxic frame, the skull was exposed and a small hole drilled ~2.5 mm posterior and ~2.5 mm lateral to bregma (*Rhabdomys*); or ~2.3 mm posterior and ~2.3 mm lateral to bregma (Mus). A 256-channel silicon probe (iridium sites, A4x64-Poly2-5mm-23s-250-177-S256, NeuroNexus Technologies, Inc., Ann Arbor, MI, USA) consisting of 4 shanks spaced 200 μm apart, each with 64 recording sites, was lowered a depth of ~3–3.5 mm into the *Rhabdomys* brain, or 2.5-3mm into the *Mus* brain, targeting the dLGN in each species. A 64-channel probe was used to record at a higher density across a larger region of the *Rhabdomys* thalamus, as shown in Figure S1 (A4x16-Poly2-5mm-23s-200-177; 4 shanks spaced 200 μm apart, with electrode sites covering 345 μm). Probes were dipped in a fluorescent dye (CM-DiI; V22888; Thermo Fisher Scientific, Waltham, MA, USA) before insertion for subsequent histological verification of recording location. Broadband neural signals were then acquired using a SmartBox recording system (NeuroNexus Technologies, Inc.), sampling at 20 kHz. Following recordings, data from each of the four electrode shanks were pre-processed by common median referencing, high-pass filtered at 250 Hz and then passed to an automated template-matching-based algorithm for single unit isolation (Kilosort;^65^). Isolated units were then extracted as virtual tetrode waveforms for validation in Offline Sorter (V3, Plexon, Dallas, TX, USA). Here, unit isolation was confirmed by reference to MANOVA F statistics, J3 and Davies-Bouldin validity metrics and the presence of a distinct refractory period (greater than 1.5 ms) in the interspike interval distribution. Spike sorted data were further analysed in MATLAB R2018a (The MathWorks).

#### Visual stimuli

Responses were recorded to a standardised set of temporally and spatially patterned monochromatic stimuli, displayed using an LCD display (width: 26.8cm height: 47.4cm; Hanns-G HE225DPB; Taipei, Taiwan) angled at ~45° from vertical and placed at a distance of ~21cm from the contralateral eye to occupy ~96° x ~63° visual angle. The temporal stimulus set consisted of a 2s step from minimum to maximum light intensity (98% contrast), followed by 2s of dark, 2s at half maximum light intensity, an 8s temporal chirp (sinusoidal modulation between dark and maximum intensity at 1-8Hz accelerating at rate of 1Hz/s), 2s at half maximum light intensity, and an 8s contrast chirp (sinusoidal modulation at 2Hz increasing from 3% to 97% contrast), as in.^15^ Spatial stimuli comprised of a sparse binary noise stimulus (5Hz, square size = 4.2°); inverting grating stimuli (1Hz) at spatial frequencies of 0.03 to 1.2 cpd, presented at two phases and four orientations (0°, 45°, 90° and 135°); and a single bar (4.2°) moving in one of 8 directions (0°, 45°, 90°, 135°, 180°, 225°, 270° and 315°) in a pseudorandom sequence. Stimulus spectra were designed to approximate the activation of each photoreceptor by natural daylight for each species (14.8 MWS-cone opsin effective photons/cm^2^/s; 12.8/12.0 SWS-cone effective photons/cm^2^/s for *Mus* and *Rhabdomys*, respectively; 14.8 rod effective photons/cm^2^/s and 14.7 melanopsin effective photons/cm^2^/s). Intensities were equivalent to those experienced on a cloudy day.

#### Analysis of dLGN responses to visual stimuli

##### Full field stimuli

Perievent spike histograms (PSTH) were generated with bin size of 30ms. Light responsive units were identified using confidence limits test based on responses to the initial 2s step of the chirp stimulus: units were classified as significant if spike firing rate during the response window was greater than 2 standard deviations above (excitation) or below (inhibition) mean firing rate during baseline window – equivalent to 95% confidence limit. ON:OFF Bias index and Sustainedness index were calculated based on previously described methods.^68,69^

To analyse temporal chirps, the mean response amplitude (maximum – minimum normalised firing rate) for each temporal frequency was fit with a half-gaussian model^16^ using least-squares minimisation to identify 5 best-fit parameters (low baseline, high baseline, gaussian spread, peak response and peak frequency).

Equation for Half Gaussians is:

$$Response\;=b_{1}+\left(a-b_{1}\right)*e^{-\left[\frac{p-w}{s}\right]\hat{2}}\;\text{for}\;w<p$$

$$Response=b_{2}+\left(a-b_{2}\right)*e^{-\left[\frac{p-w}{s}\right]\hat{2}}\;\text{for}\;w>p$$

Where *w* is the temporal frequency (Hz), *p* is the temporal frequency (TF) that produces peak response, *a* is the maximum response amplitude at optimum TF, *s* is the Gaussian spread, *b1* is the baseline for frequencies lower than peak TF, *b2* is the baseline for frequencies greater than the peak TF. Peak temporal frequency was rounded to nearest integer to address the limited resolution of temporal frequency analysis (sample every 1Hz).

For contrast chirps, the response amplitude (maximum – minimum normalised firing rate during each period of the contrast chirp stimulus) was normalised to baseline activity (1s before contrast chirp onset) for each unit. This was plotted against Michaelson contrast and then fit using a Naka-Rushton curve using least-squares minimisation to identify 4 best-fit parameters (top, bottom, C50 and slope).

Equation for Naka-Rushton curve is

$$\text{Response}\;=\;\text{Bottom}\;+(\text{Top}\;*\frac{C^{n}}{C^{n}+C50^{n}})$$

Where *n* = slope, *C* = Michelson contrast and C50 is contrast that produces half maximum response. C50 was constrained between 0 and 1, and slope was constrained between 0 and 10.

##### Functional clustering

Sparse Principal components were generated for the full-field temporal stimulus using the SPaSM toolbox,^64^ as described in Baden et al.^15^ This allows the extraction of response features that are localised in time. We pooled mean PSTH (25ms bins) for all light responsive units from both groups and extracted up to 30 features with 5 non-zero time bins. We then discarded those that accounted for < 1% of the variance. Response features that met these criteria for each window were then combined to produce a total of 30 features for dLGN. sPCs from integrated *Mus* and *Rhabdomys* data were then clustered with a mixture of Gaussian models, a probabilistic model using random initialisation. The optimum number of clusters was determined based on the lowest Bayesian information criteria, which rewards fit but penalises complexity, and a Bayes factor below 6 as a threshold for when there was no longer evidence for further splitting.

To compare distribution of units across clusters between two groups, we calculated the distance (Euclidian norm of the difference in the mean relative proportion of neurons in each cluster) between *Mus* and *Rhabdomys* data and compared this with a null distribution obtained by randomly shuffling retinal recordings between two groups 10,000 times. To compare proportion of units from each group within each cluster, we first calculated the % of total units from each group for each recording and then found the difference between mean of *Mus* and *Rhabdomys*. This was compared with a null distribution generated as above.

##### Receptive field mapping

The spatio-temporal receptive field was derived for each unit by generating the spike triggered average (STA) of responses to a sparse binary noise stimulus (5Hz, square size = 4.2°). The separable spatial and temporal components where then extracted from the raw STA matrix by finding the signal peak. RF locations and sizes were then generated by fitting spatial receptive fields with 2D Gaussian function (using lsqcurvefit function, MATLAB). The receptive field size for individual cells was the average of the standard deviation of Gaussians fitted to each dimension. Temporal receptive fields were generated by plotting the temporal response of the RF centre. Receptive field overlap was calculated as a percentage overlap of the 2D Gaussian for pairs of receptive fields recorded in the same animal.

##### Spatial frequency tuning and spatial summation

To assay changes in spatial frequency tuning, inverting gratings (Michelson contrast between dark and light bars = 98%) were presented in 4 orientations at two phases (phase shifted 90°), at 5 different spatial frequencies (0.03 to 1.2 cpd) at 1Hz. For each unit, response amplitudes were quantified (relative to pre-stimulus firing) for each phase/orientation combination for each spatial frequency to determine the optimal stimulus (R_pref_). R_orth_ was the response to stimuli presented at 90° to the preferred orientation. The orientation selectivity index (OSI) was calculated as the ratio of (R_pref_-R_orth_)/(R_pref_+R_orth_) at the preferred spatial frequency. Cells exceeding an OSI of 0.5 were classed as ‘orientation selective’.

Response linearity was evaluated by quantifying the firing rate of units in response to stimuli that were larger than the calculated receptive field size for an individual unit. Continuous firing rates during the stimulus presentation were Fourier analysed to extract amplitudes of the first and second harmonic components (F1 and F2, at 1Hz and 2Hz), at the preferred and null (90° phase shifted) stimulus (F1_pref_, F1_null_, F2_pref_, F2_null_). A linearity index (LI) of the response was then calculated as F1/F2 for both preferred and null phases, whereby a LI<1 describes a dominant F2 amplitude, indicating nonlinear spatial summation.

##### Motion selectivity

A single drifting bar (4.2°; Michelson contrast= 98%) was presented on a screen, moving in one of 8 directions (0°, 45°, 90°, 135°, 180°, 225°, 270° and 315°) in a pseudorandom order. For each unit, response amplitudes were quantified during the presentation of movement (relative to pre-stimulus baseline) for each direction of motion. Since the location of the stimulus relative to the location of a unit’s receptive field was not precisely known, to explore the kinetics of responses, a STA was generated (as described above) for the preferred direction of motion. For presentation purposes, responses were clustered using a Kmeans cluster (MATLAB) finding 3 response clusters.

Two methods were used to explore direction selectivity. First, the mean and variance of circular data were computed using the CircStat, a MATLAB toolbox,^63^ to describe the angle and magnitude of directional selectivity. A direction selectivity index was also calculated as the ratio of (Rpref-Rnull)/(Rpref+Rnull), where Rpref was the direction of motion at which the maximum evoked response occurred, and Rnull was response to movement in the opposite direction to this. Cells exceeding a direction selectivity index of 0.33 were classed as ‘direction selective’.

#### Behavioral measurement of spatial acuity

Behavior was measured in adult *Mus* (3 months) and *Rhabdomys* (aged 5–8 months). Animals were placed into an open field arena as described in.^19^ The arena was illuminated with an infrared light, with ongoing behavior tracked using a programmable global shutter camera (Chameleon 3; Point Grey) fitted with infrared cut-on filter (Edmund Optics). Visual stimuli were delivered using a monochromatic projector projecting onto a rear projection screen. Experiments were controlled using Psychopy (verison v2021.2.3^62^). Frame acquisition was synchronized with the projected images via a common electrical trigger delivered by an Arduino Uno board (arduino.cc) controlled by Psychopy. Triggered acquisition through external TTL from the Arduino board was enabled on Chameleon 3 cameras through FlyCapture2 software (Point Grey).

Animals were all habituated to the behavioral arena for 15 minutes the day prior to the stimulus presentation (testing) session. During the testing session, animals were placed into the arena with overhead screen showing a uniform grey background. After an acclimatisation period of 6 minutes, a looming stimulus was presented as a disc against this grey background, which rapidly widened (over 200ms) to cover 37.5° of visual space, and remain on the screen for a following 1s. The disc was either fully black (as frequently used in such paradigms^70^), or a sinusoidal grating which was isoluminant with the background, with spatial frequency decreasing with each presentation (moving from 2.9 to 0.1 cpd). Looming stimuli appeared with an interval of 1 minute,

Movement was analyzed using custom MATLAB scripts that detected the location of the animal in each frame, as described in.^71^ We calculated the animal’s movement from frame to frame, and detected flight or freeze responses in a similar approach to described in ^70^: flights were defined as periods when mouse speed exceeded baseline activity by more than 3 standard deviations; freezes were defined as periods of time during which no movement was detected for at least 1s. The first spatial frequency to which animals responded with a freeze/flight was compared between species.

#### Electroretinography

ERGs were recorded in adult *Mus* (aged 4-5 months) and adult *Rhabdomys* (aged 7-8 months). All ERGs were recorded at subjective midday following dark adaptation for a period of 6 hours. Animals were anaesthetised under isoflurane in a 95/5% Oxygen/CO2 mix at a flow rate of 0.5 – 1.0L/min. Isoflurane concentrations of 5% and 1.5-3.5% were used for induction and maintenance of anaesthesia, respectively. A topical mydriatic (tropicamide 1%; Bausch and Lomb) and hypromellose eye drops were applied to the recording eye before placement of a corneal contact-lens–type electrode. A needle reference electrode (Ambu, Neuroline) was inserted approximately 5mm from the base of the contralateral eye, and a second subcutaneous needle in the scruff acted as a ground. Electrodes were connected to a Windows PC via a signal conditioner (Model 1902 Mark III, CED) that differentially amplified (X3000) and filtered (band-pass filter cut off 0.5 to 200Hz) the signal, and a digitizer (Model 1401, CED). Core body temperature was maintained at 37°C throughout recording with a homeothermic heat mat (Harvard Apparatus).

Visual stimuli were generated with a combination of violet, blue and cyan elements of a multispectral LED light source (Lumencor). Intensities were modulated via pulse width modulations via an Arduino Uno. Light from the light engine passed through a filter-wheel containing neutral-density filters (reducing the light by between 10^1^ and 10^5^) and focused onto opal diffusing glass (5mm diameter; Edmund Optics Inc.) positioned <5mm from the eye. All LED intensities were controlled dynamically with a PC. Stimuli were measured at the corneal plane using a spectroradiometer (SpectroCAL II, Cambridge Research Systems, UK) between 350–700nm. Dark-adapted stimuli were presented as a 10ms flash of stimulus spectra from background across a range of 9.1 to 15.8 photons/cm^2^/s (interstimulus intervals ranging from 1-6s with increasing intensities). Light-adapted stimuli were presented as square-wave modulations from background at 80.5% Michelson contrast at 2Hz at a background of 14.6 log photons/cm^2^/s, after 15 minutes background adaptation. ERG responses were analysed in MATLAB. For flash responses, a-wave amplitude was calculated relative to baseline prior to stimulus onset, with b-wave with reference to the a-wave trough. For step responses, b- and d-waves were calculated relative to preceding a-wave.

#### Immunohistochemistry

Retinal wholemounts were initially permeabilized in a 1% TritonX-100 solution in PBS (1% PBS-X; PBS–Phosphate-buffered saline; 3 ×10 mins) before being blocked in a mixture of 10% goat serum (Sigma Aldrich, United Kingdom) in 0.2% PBS-X for 3 h at room temperature. Tissue was subsequently incubated with primary antibody rabbit anti-melanopsin antibody (UF006, Advance Targeting Systems, 1:2000) to label ipRGCs for 3 days at 4°C. After this, wholemounts were washed thoroughly in 0.2% PBS-X before being incubated in the secondary antibody Alexa-546 conjugated goat anti-rabbit (1:500; Life Technologies), for 12 h at 4°C. The tissue was then thoroughly washed in 0.2% PBS-X before undergoing one final wash in dH_2_0 and mounted.

Retinal sections were initially permeabilized in a 0.2% TritonX-100 solution in PBS (0.2% PBS-X; PBS–Phosphate-buffered saline; 3 x 10 mins) before being blocked in a mixture of 10% goat serum (Sigma Aldrich, United Kingdom) in 0.2% PBS-X for 1 h at room temperature. Tissue was subsequently incubated with primary antibody rabbit anti-PKCα (ab32376, Abcam, 1:1000) to label rod bipolar cells for 2h at room temperature. After this, sections were washed thoroughly in PBS 0.05% tween (4 x 10 mins) before being incubated in the secondary antibody Alexa-546 conjugated goat anti-rabbit (1:500; Life Technologies), for 2h at room temperature. The sections were then thoroughly washed in 0.2% PBS-X before undergoing one final wash in dH_2_0 and mounted.

#### Fluorescent *in situ* hybridization chain reaction

*Mus* (n=3) and *Rhabdomys* (n=3) retinas were dissected and used for HCR™ RNA-FISH (Molecular Instruments) according to the manufacturer protocol for fixed wholemount tissue. Briefly, retinas underwent a series of dehydration and rehydration steps (75%, 50%, 25% methanol solution) and then treated with proteinase K (10 μg/mL). Retinas were pre-hybridized with probe hybridization solution and incubated overnight in probe solution containing custom ordered probes *Mus* or *Rhabdomys Opn4* (2 pmol). Retinas were pre-amplified in amplification buffer and incubated overnight in amplification solution containing hairpins H1 and H2 (30 pmol each; amplification fluorophore 594). Retinas were washed in sodium chloride sodium citrate tween 20 solution and stored at 4°C before imaging. Two *Rhabdomys* retinas were labelled with custom ordered probes targeting *Rhabdomys Opn4* and *Eomes*, using amplification fluorophores 594 and 647, respectively. Cells were detected using QuPath^72^ cell detection feature.

Following immunohistochemistry/HCR-FISH, high magnification images of retinal wholemounts and retinal sections were collected using a Leica SP8x AOBS Inverted confocal using a 40x / 0.85 HCX PL APO objective. The confocal settings were as follows, pinhole [1 airy unit], scan speed [600Hz unidirectional], format [1024 x 1024]. Images were collected using hybrid detectors with the following detection mirror settings; [572-600nm] using the [white light laser with 560nm (10%)] laser line. When acquiring 3D optical stacks, the confocal software was used to determine the optimal number of Z sections. The maximum intensity projections of these 3D stacks were used for further quantification and are those shown in results. Low magnification images of wholemount retinas were imaged with an Axio Imager D2 upright microscope and captured using a Coolsnap Hq2 camera (Photometrics) through Micromanager software v1.4.23.

#### Analysis of transcriptomic datasets

##### Alignment and quantification of gene expression

Preprocessing of raw sequencing data was performed using Cellranger (v6.2, 10X Genomics). Sequencing reads were demultiplexed using “cellranger mkfastq” to obtain a separate set of fastq.gz files for each of the 7 samples. These files were then aligned to a reference genome^13^ using “cellranger count” with the –include-introns flag to include both exonic and intronic reads, resulting in a gene expression matrix (GEM) summarizing transcript counts within each sample. GEMs from each of the 7 samples were combined (column-wise concatenated) to yield a total GEM.

##### Segregation of major retinal cell classes

Analysis of the total GEM was performed in R, with the workflow based on Seurat v4.3.0 for single-cell analysis developed and maintained by the Satija laboratory^66^ (https://satijalab.org/seurat/). Transcript counts in each cell were normalized to a total library size of 10,000 and log-transformed (X → log (X + 1)). We identified the top 2,000 highly variable genes and applied principal component analysis (PCA) to obtain a linear factorization of the submatrix corresponding to these highly variable genes. Using the top 20 principal components for each cell, we built a k-nearest neighbor graph on the data, and then clustered with a resolution parameter of 0.5 using Seurat’s FindClusters function. Each cluster of cells was assigned to a retinal cell class based on canonical markers characterized in mice^26^; for example, *Vsx2, Otx2* and *Grik1* were used to identify bipolar cells, and *Rbpms, Nefl* and *Nefm* were used to identify RGCs.

##### Supervised classification analysis

Supervised classification analysis was used to explore transcriptional correspondence between *Rhabdomys* and *Mus* types. RGCs were separated from the *Rhabdomys* total GEM and, as the *Rhabdomys* genome was annotated with mammalian orthologs across murid genome assemblies, were merged with a reference *Mus* RGC atlas^25^ using genes that were present in both the *Rhabdomys* GEM and *Mus* reference GEM to create a joint GEM. We used the Canonical Correlation Analysis-based framework in Seurat, integrating by species of origin, to produce an integrated version of the joint GEM adjusted to account for species specific differences in gene expression. The top 2000 variable features of the integrated GEM were used to train a gradient boosted decision tree using the reference *Mus* RGC cells, implemented in R using the xgboost package.^67^ The *Mus* RGC-trained classifier was used to assign a *Mus* identity to each *Rhabdomys* RGC based on its expression of the 2000 training features. To identify how rare *Mus* RGC types mapped to *Rhabdomys* types, a similar decision tree model was trained using the *Rhabdomys* RGC cells and applied to *Mus* RGCs to assign a *Rhabdomys* identity to each *Mus* RGC. Similarly, this was done with *Rhabdomys* bipolar cells with a relevant *Mus* bipolar cell atlas to assign *Mus* bipolar labels to each *Rhabdomys* bipolar cell, and vice versa. These reciprocal mappings helped us validated the robustness of the label transfer procedure.

To summarize the results of the supervised classification mapping, we calculated a modified Jaccard index for each pair of *Rhabdomys* and assigned *Mus* types. For a given *Rhabdomys* type A and assigned *Mus* type B, we calculated

$$\text{J}(\;\text{A},\;\text{B})=\frac{\left|A\cap B\right|}{\min(|\text{A}|,|\text{B}|)}$$

### Quantification and Statistical Analysis

#### Statistical analysis and software used

Statistical details of experiments can be found in figure legends and figures, as well as associated results text. This includes the statistical tests used, n number (animals and/or neurons), and a description of centre and dispersion (typically mean +/- SEM). A p value of 0.05 was used to determine significance. No randomisation or stratification methods were required (comparisons were solely made between species). No data were excluded from the analysis, other than neurons deemed to be non-light responsive/outside of the region defined as the dLGN based on histological verification of recording sites. Data were tested for normality, and parametric/ non-parametric statistical tests were performed accordingly.

## Supplementary Material

**Supplemental Information**

Supplemental information can be found online at https://doi.org/10.1016/j.cub.2025.02.014.

Supplementary Information

## In brief

Allen et al. apply a systems-level transcriptional and functional classification of neurons in the visual system of related murids: nocturnal *Mus musculus* and day-active *Rhabdomys pumilio*. They demonstrate how realignments in the visual code can reflect selective expansion of retinal cell types in each species.

## Highlights

- The visual code is qualitatively distinct in nocturnal *Mus* vs. diurnal *Rhabdomys*
- *Rhabdomys* visual code is biased toward non-linear spatiotemporal summation
- Retinal BC and RGC subtypes supporting non-linear responses are expanded in *Rhabdomys*
- Visual ecology shapes the retinal cell composition and the visual code in murids
