# Supplementary material for: Altered proportions of retinal cell types and distinct visual codes in rodents occupying divergent ecological niches
Source: Curr Biol. Author manuscript; Available in PMC 2026 Aug 6. (PMC7619322; doi:10.1016/j.cub.2025.02.014)
Supplement: Supplementary file 1 [file EMS216069-supplement-Supplementary_Information.pdf]

Current Biology, Volume *35*

Supplemental Information

Altered proportions of retinal cell types and distinct visual codes in rodents occupying divergent ecological niches

Annette E. Allen, Joshua Hahn, Rose Richardson, Andreea Pantiru, Josh Mouland, Aadhithyan Babu, Beatriz Baño-Otalora, Aboozar Monavarfeshani, Wenjun Yan, Christopher Williams, Jonathan Wynne, Jessica Rodgers, Nina Milosavljevic, Patrycja Orlowska-Feuer, Riccardo Storchi, Joshua R. Sanes, Karthik Shekhar, and Robert J. Lucas

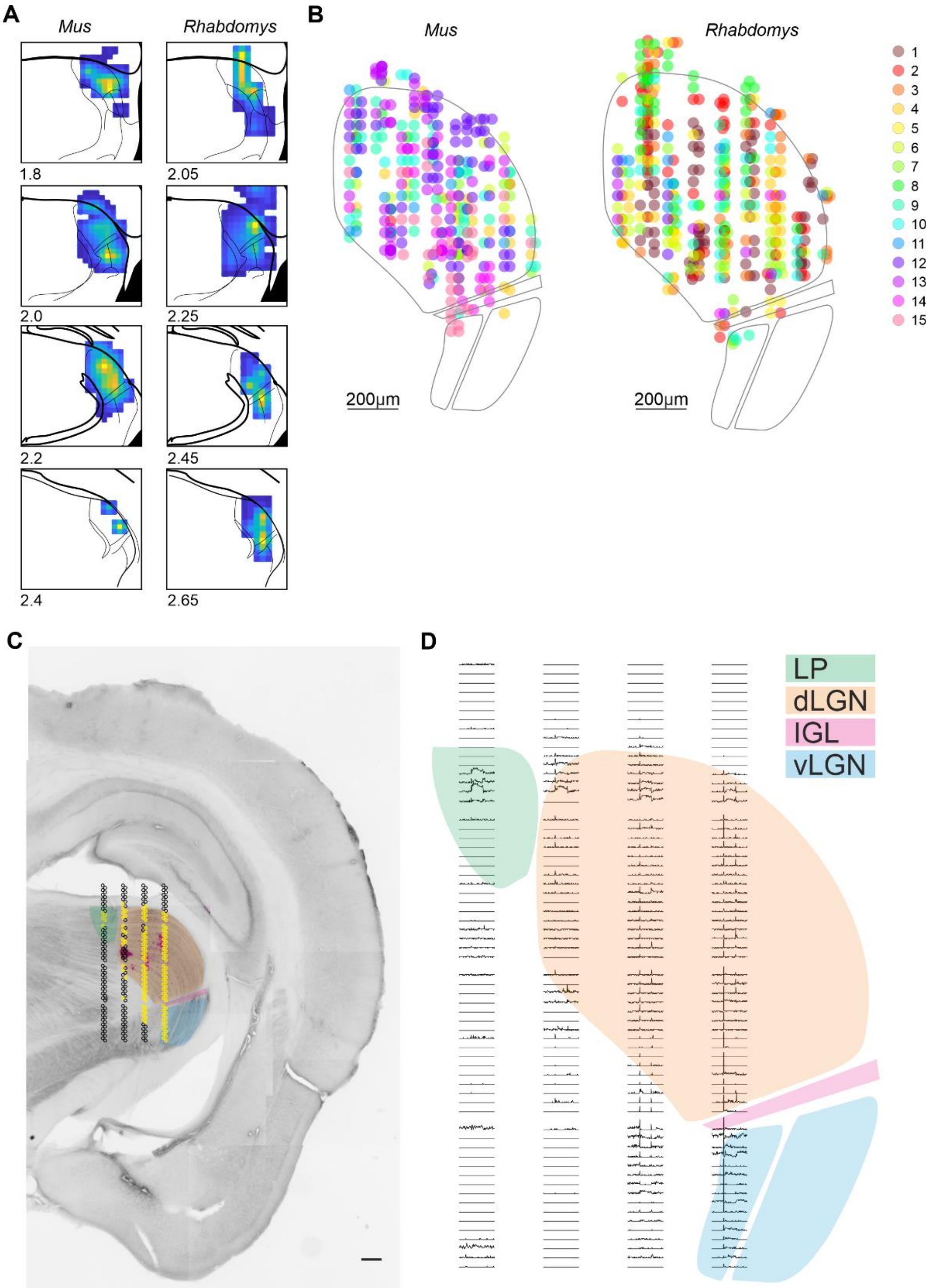

Figure S1. Location of recorded units in *Mus* and *Rhabdomys.* Related to Figure 1
A) Heat map showing reconstruction of light-evoked responses in *Mus* (left) and *Rhabdomys* (right). Histological location of dLGN is approximated in *Rhabdomys* from a scaled version of *Mus* stereotaxic atlas. Numbers below plots indicate distance from bregma. **B)** Location of units assigned to each cluster for *Mus* (left) and *Rhabdomys* (right). For simplicity, location is compressed on rostro-caudal axis and superimposed onto dLGN map in coronal plane. Colour code for each cluster number is shown to right. **C)** Histological reconstruction of recording sites from one representative *Rhabdomys.* Green fluorescence indicates electrode location. Predicted location of individual electrode sites are indicated in black (no light response detected) or yellow (light response detected). Location of regions in lateral thalamus are highlighted, with green indicating LP (lateral posterior thalamic nucleus); orange indicating dLGN; pink indicating IGL (intergeniculate leaflet) and blue indicating ventral lateral geniculate nucleus (vLGN). Scale=200µm. **D)** Magnified version of **C)**, with example response to 2s light step plotted at electrode location (normalised to maximum firing rate). Brain regions coloured as previous.

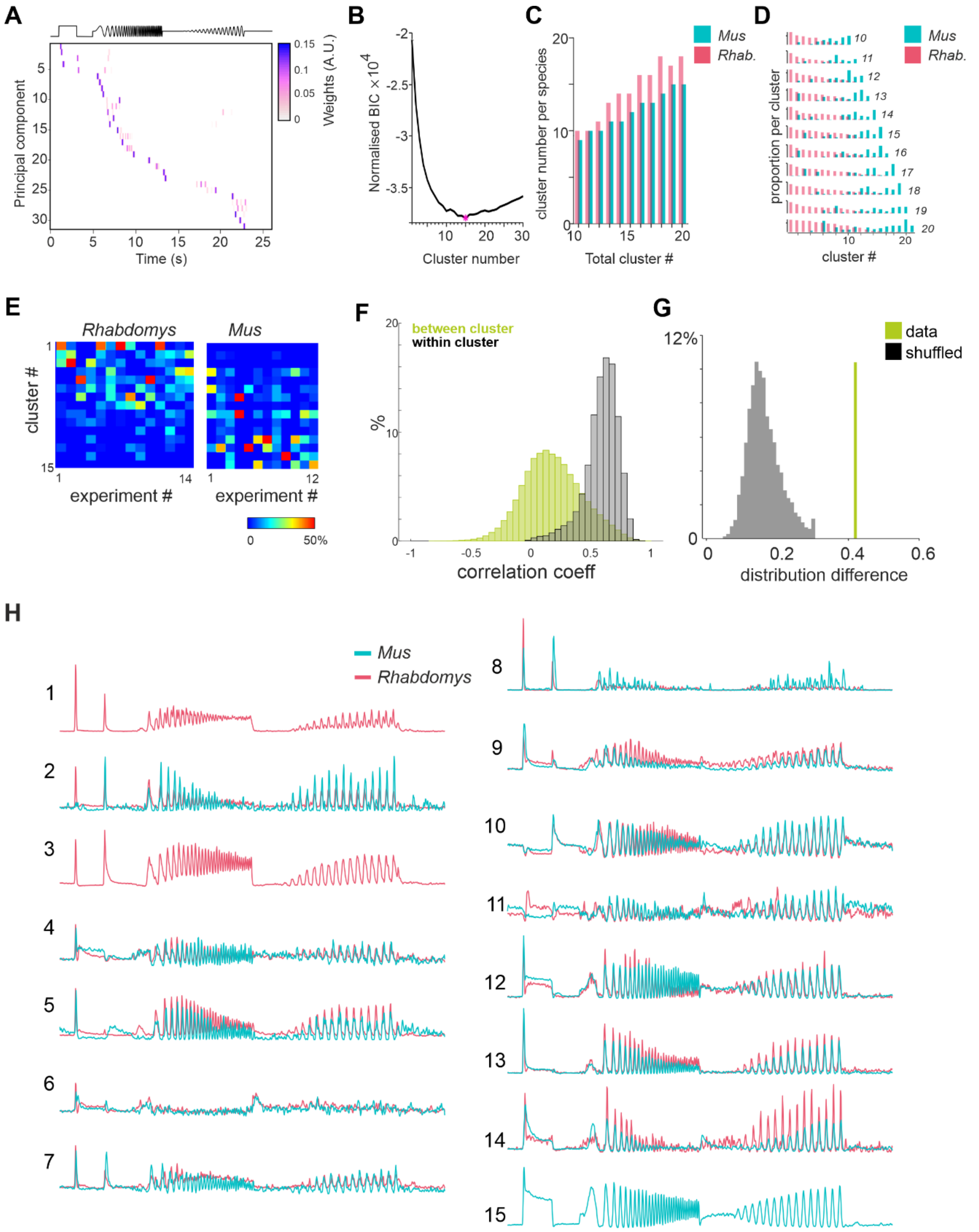

Figure S2. Clustering of visually evoked responses in *Mus* and *Rhabdomys*. Related to Figure 1

A) Heatmap showing weights of the principal components from sparse principal component analysis of *Mus* and *Rhabdomys* dLGN unit firing rates in response to temporally modulated stimulus. Stimulus profile is shown in upper panel. Rows of heatmap depict the sparse principal components contributing to >1% variance, organised according to time of peak. **B)** Normalized Bayesian information criterion (BIC) curve; pink point indicates the optimal numbers of clusters (15). **C&D)** Number (C) and distribution (D) of clusters assigned to each species when cluster number is fixed (ranging from 10-20 clusters). Note that irrespective of cluster number, the difference among species remains consistent. *Rhabdomys* in red and *Mus* in cyan. **E)** Heatmap showing percentage of responses from each cluster as a function of experiment (14 recordings in *Rhabdomys*; 12 recordings in Mus). **F)** Histogram showing correlation between *Mus* and *Rhabdomys* cluster PSTHs within clusters (black) and between clusters (green). **G)** Euclidian difference in cluster distribution between species (green line), showed alongside histogram of null distribution of shuffled experiments (p=0). **H**) Mean response profile of each cluster, separated into *Rhabdomys* (red) and *Mus* (cyan).

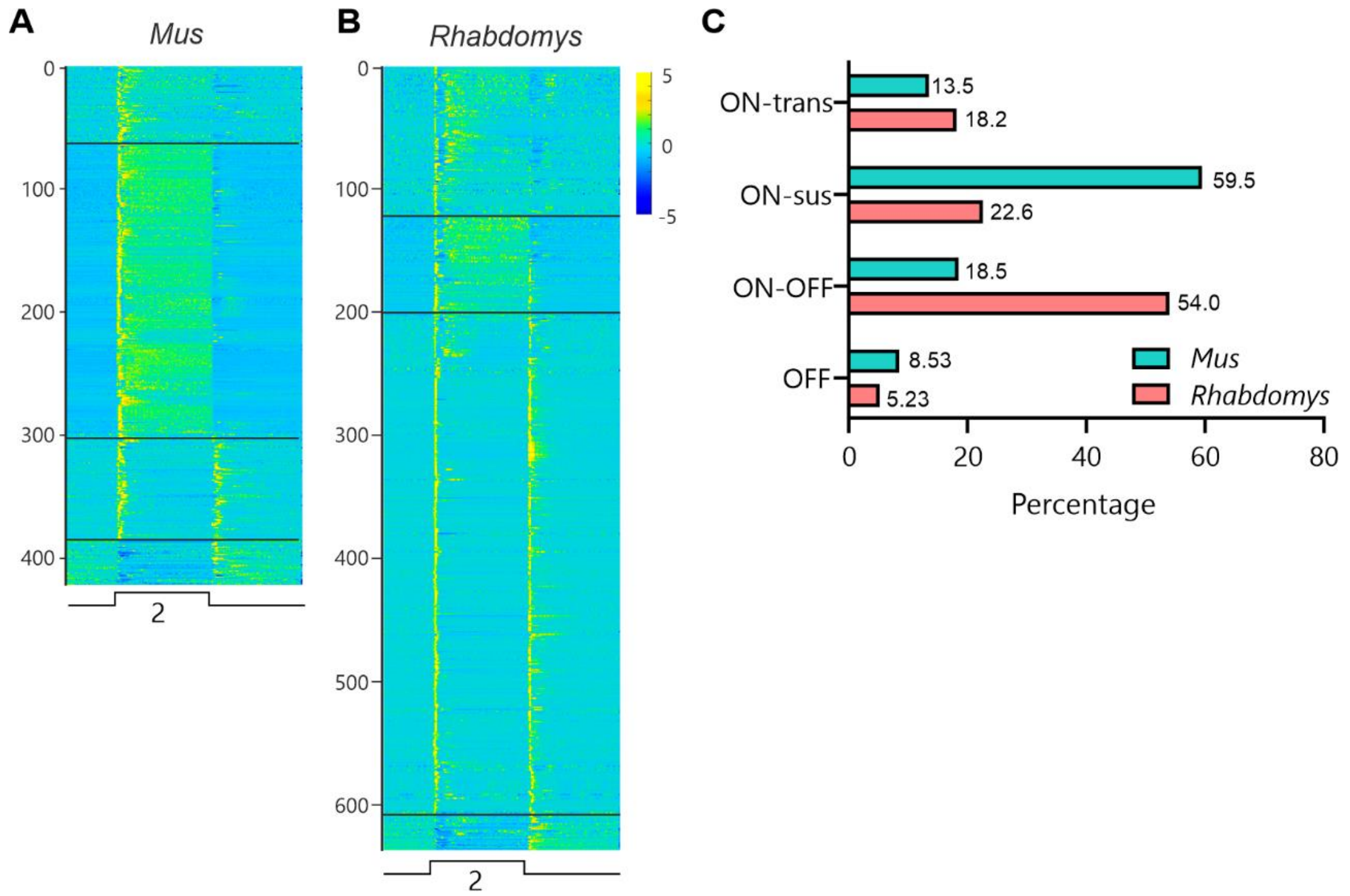

Figure S3. Responses to a 2s full field light step in *Mus* and *Rhabdomys.* Related to Figure 1
A&B) Heat map showing mean light response in response to a 2s full-field step (97% Michelson contrast) for responsive neurons in *Mus* (A) and *Rhabdomys* (bB), ordered as a function of response type (transient ON, sustained ON, on-off, off sustained). **C)** Bar graph showing proportion of units categorised into one of four response types: transient ON; Sustained ON; ON-OFF; OFF in *Mus* and *Rhabdomys.*

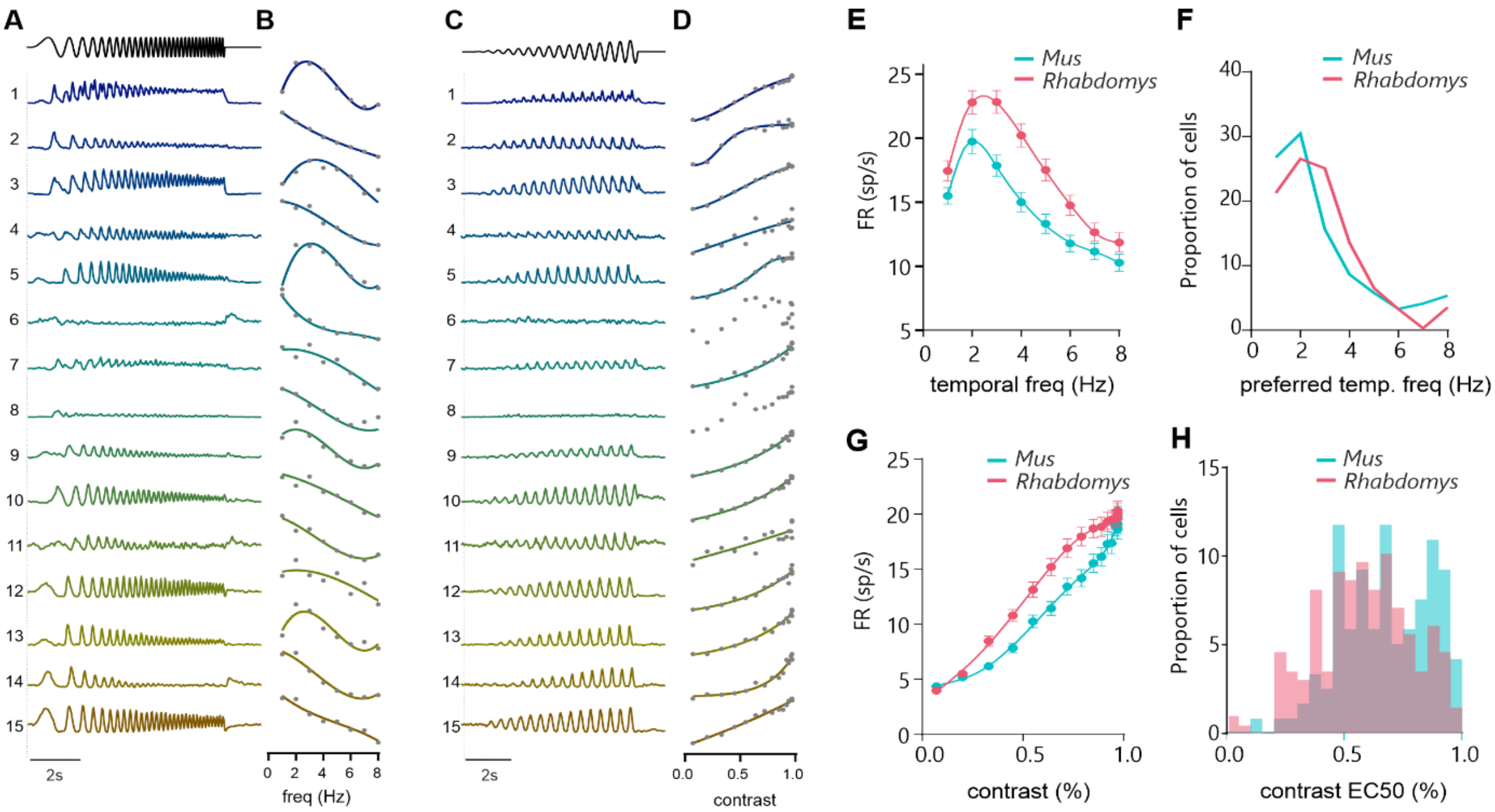

Figure S4. Temporal and contrast sensitivity of *Mus* and *Rhabdomys.* Related to Figure 1

A) Mean response profile of clusters 1-15 to increasing frequency sinusoidal stimuli. **B)** Response amplitude to increasing frequency sinusoidal stimulus, binned at a resolution of 1Hz, for individual clusters. Data shows mean, normalised response profile. **C)** Mean response profile of clusters 1-15 to increasing contrast sinusoidal stimulus. **D)** Response amplitude to increasing contrast sinusoidal stimulus, for individual clusters. Data shows mean, normalised response profile. **E)** Response amplitude to increasing frequency sinusoidal stimulus, binned at a resolution of 1Hz, for *Mus* (cyan) and *Rhabdomys* (red). Data shows mean+/- SEM. **F)** Distribution of preferred temporal frequency for *Mus* (cyan) and *Rhabdomys* (red). **G)** Response amplitude to increasing contrast sinusoidal stimulus, shown for *Mus* (cyan) and *Rhabdomys* (red); data shows mean+/- SEM. **H)** Distribution of C50 values (half-maximum of Naka-Ruston curve) for *Mus* (cyan) and *Rhabdomys* (red).

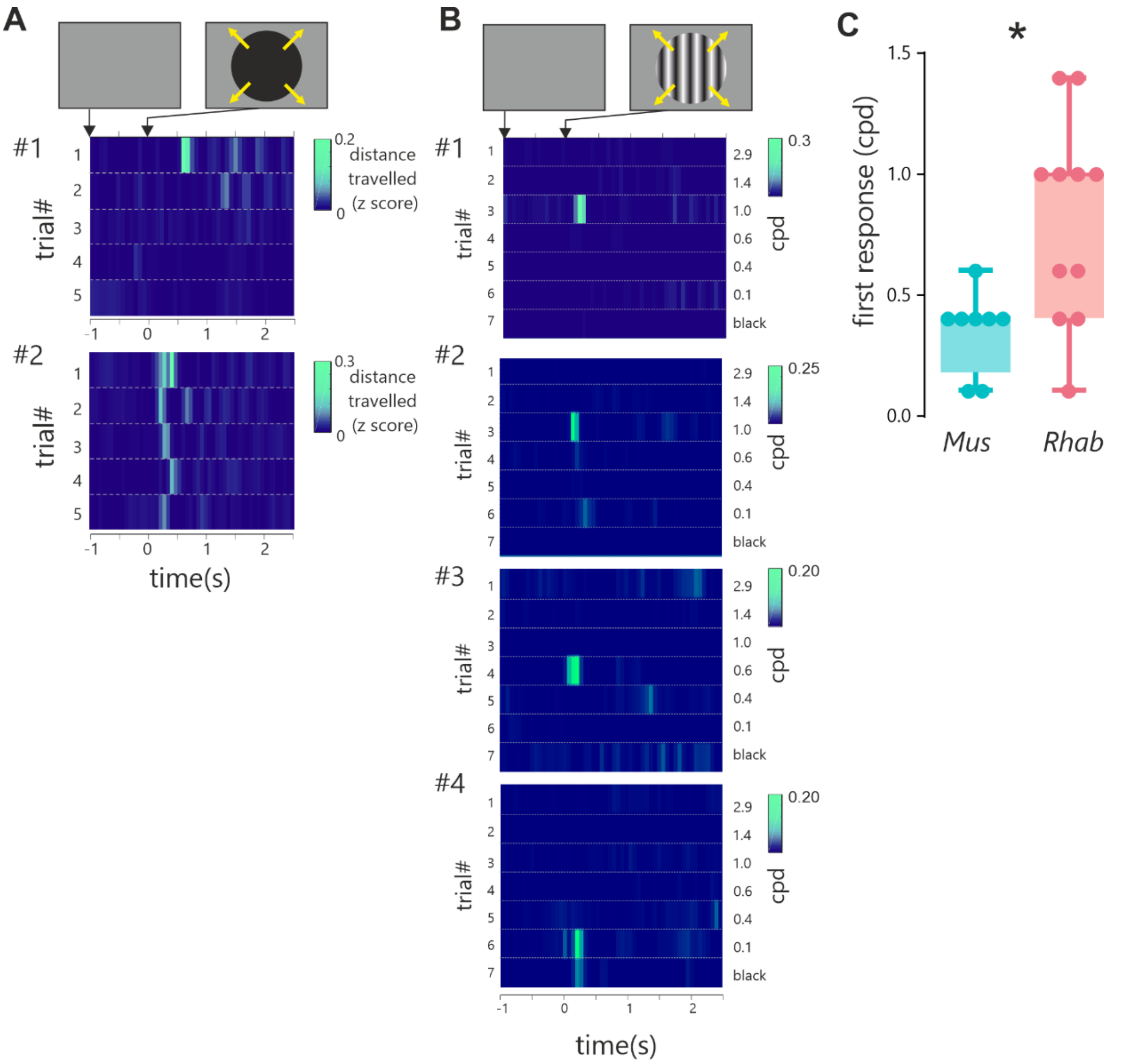

Figure S5. Behavioural assay of spatial vision reveals that *Rhabdomys* can respond to spatial frequencies beyond the established maximum spatial resolution of *Mus* vision. Related to Figure 2.
S) Locomotor response of *Rhabdomys* to looming black disc. Upper panel shows timing of stimulus presentation (black looming disc). Lower panels show two representative responses from individual *Rhabdomys* to this presentation. Example #1 shows a response that was typical of most animals tested, where a brief motor response ('leap') was apparent in <1s following the first stimulus presentation (median response latency = 0.265ms, n=5). There was no response to subsequent stimuli. Example #2 shows the only animal that showed responses to each stimulus presentation and is included here to demonstrate the specificity of this motor response to the stimulus. **B)** Locomotor response of *Rhabdomys* to looming grey-scale sinusoidal gratings at increasing spatial frequency. Upper panel shows cartoon of looming grating stimulus appearing at time 0. Lower panels show change in activity (normalised distance travelled) for four representative *Rhabdomys*. Each plot shows activity in response to each spatial frequency presented in sequence from 2.9-0.1 cpd (final stimulus ('black') was a fully black disc). Note that typically only one stimulus evoked a significant response (e.g. 1 cpd in example #1), with subsequent stimuli driving a minimal change in behaviour. **C)** Grating spatial frequency at which response first observed for individual *Mus* (cyan) and *Rhabdomys* (red), with box and whiskers highlighting median, 25th and 75th percentiles, min and max. Frequency at first response was significantly different between species (Mann Whitney test, p=0.014; n=8 *Mus*, n=11 *Rhabdomys*). The mean frequency of first response in *Mus* matches estimates of acuity produced by other methods in this species[S1].

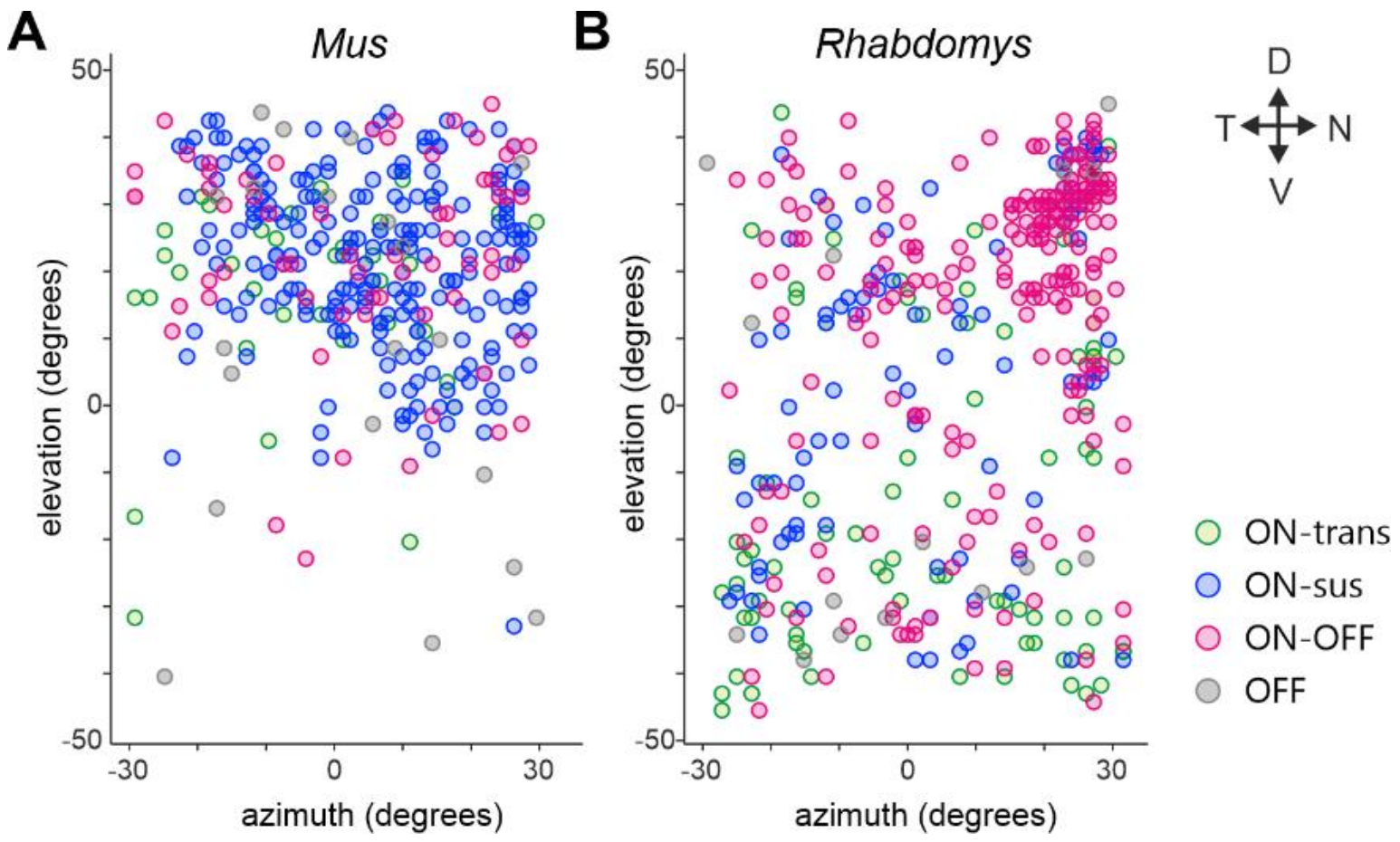

Figure S6. Receptive field locations of units recorded in *Mus* and *Rhabdomys* dLGN. Related to Figure 1.

Scatter plots showing location of receptive field centres recorded in *Mus* (**A**) *and Rhabdomys* (**B**) dLGN in response to spatiotemporal binary noise stimulus. Dots are colour coded according to basic response type (ON-transient, ON-sustained, ON-OFF and OFF). Receptive fields were only mapped in a proportion of dLGN units, and there was a tendency for receptive fields to be in the upper/nasal region of visual space covered by the display. n = 346 and n = 419 units in *Mus* and *Rhabdomys,* respectively.

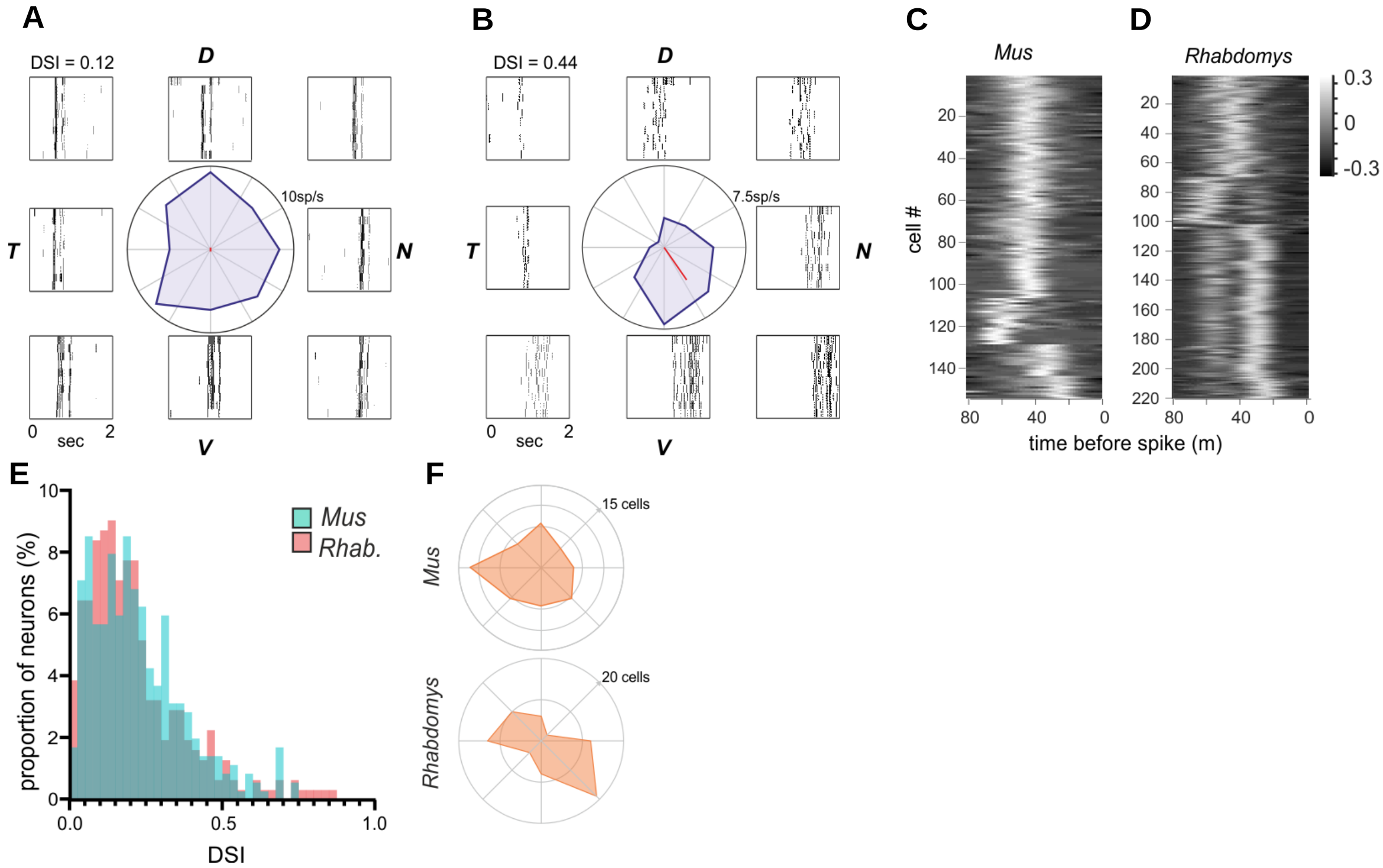

Figure S7. Direction selectivity and responses to motion. Related to Figure 2

A&B) Representative neurons from *Rhabdomys* with low (DSI = 0.12) or high (DSI = 0.44) DSI values. Polar plot in centre plots change in firing rate in each direction of motion, and rasters plots to side showing the response to repeated stimuli at each direction. Dorsal, ventral, nasal and temporal locations indicated by D, V, N and T. **C&D)** Heat maps show spike triggered average of luminance changes in the RF centre, as a function of time (-80ms – 0), for all neurons in which we were able to map receptive fields, in *Mus* (C) and *Rhabdomys* (D). **E)** histogram of DSI values for *Rhabdomys* (red) and *Mus* (cyan) dLGN neurons (Kolmogorov-Smirnov test: P=0.193) **F),** distribution of tuning preference for DS neurons in *Mus* (top) and *Rhabdomys* (bottom) with DSI > 0.33.

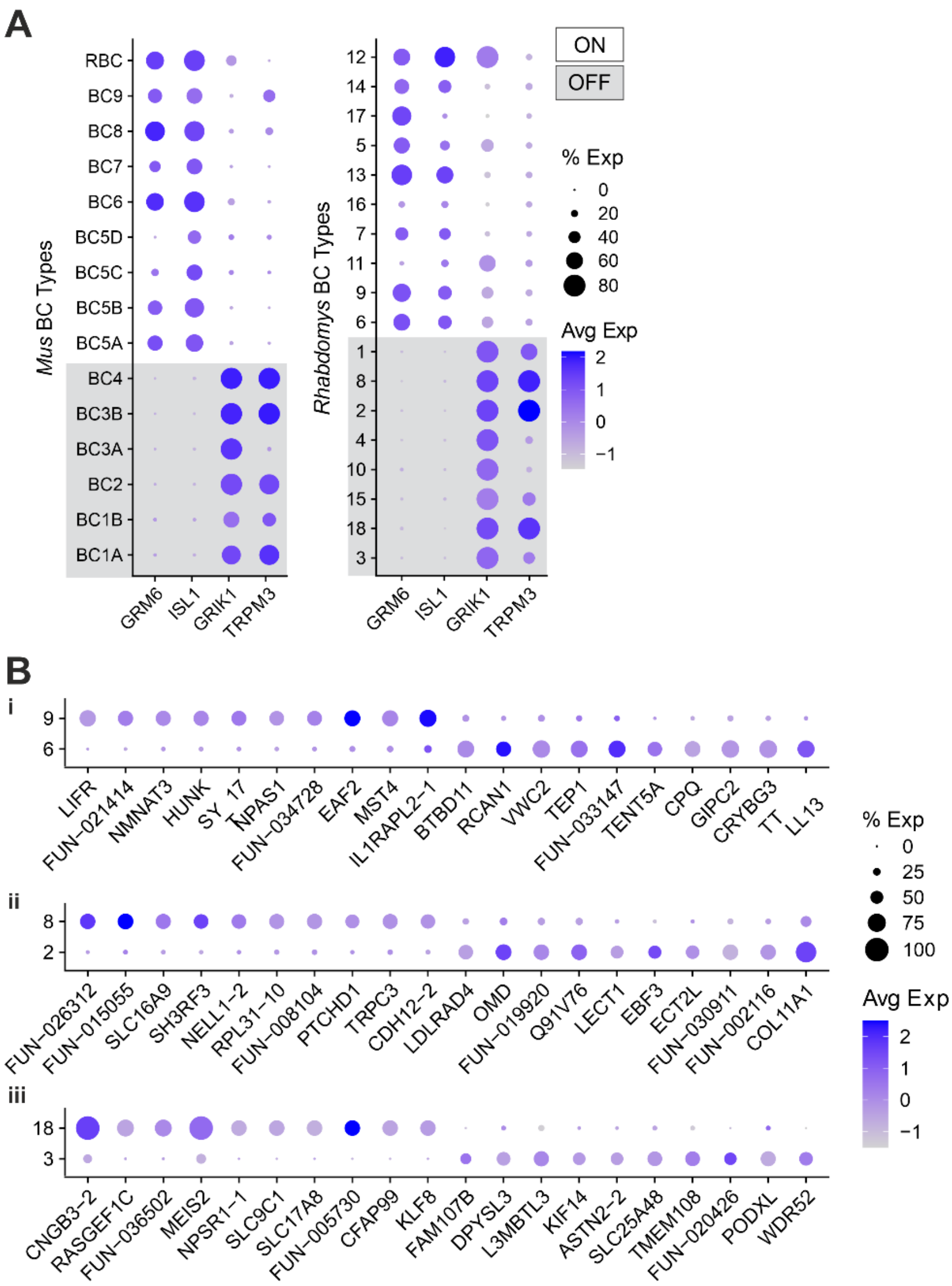

Figure S8. Gene expression profiles of Rhabdomys bipolar cell pairs. Related to Figure 5.
A) Dotplots showing genes (columns) that distinguish OFF and ON BC types (rows) as described in [S1]. *Mus* data is shown to left and *Rhabdomys* to right. The size of each circle represents the percentage of cells within each type that express the given gene, and colour represents the average expression of that gene within the cluster. Grey shading highlights BC types mapping to OFF types. **B)** Dotplots showing genes (columns) that distinguish pairs (i-iii) of *Rhabdomys* BC types (rows) that map to the same *Mus* BC type. The size of each circle represents the percentage of cells within a type that express the given gene, and the color represents the average expression of that gene within that cluster.

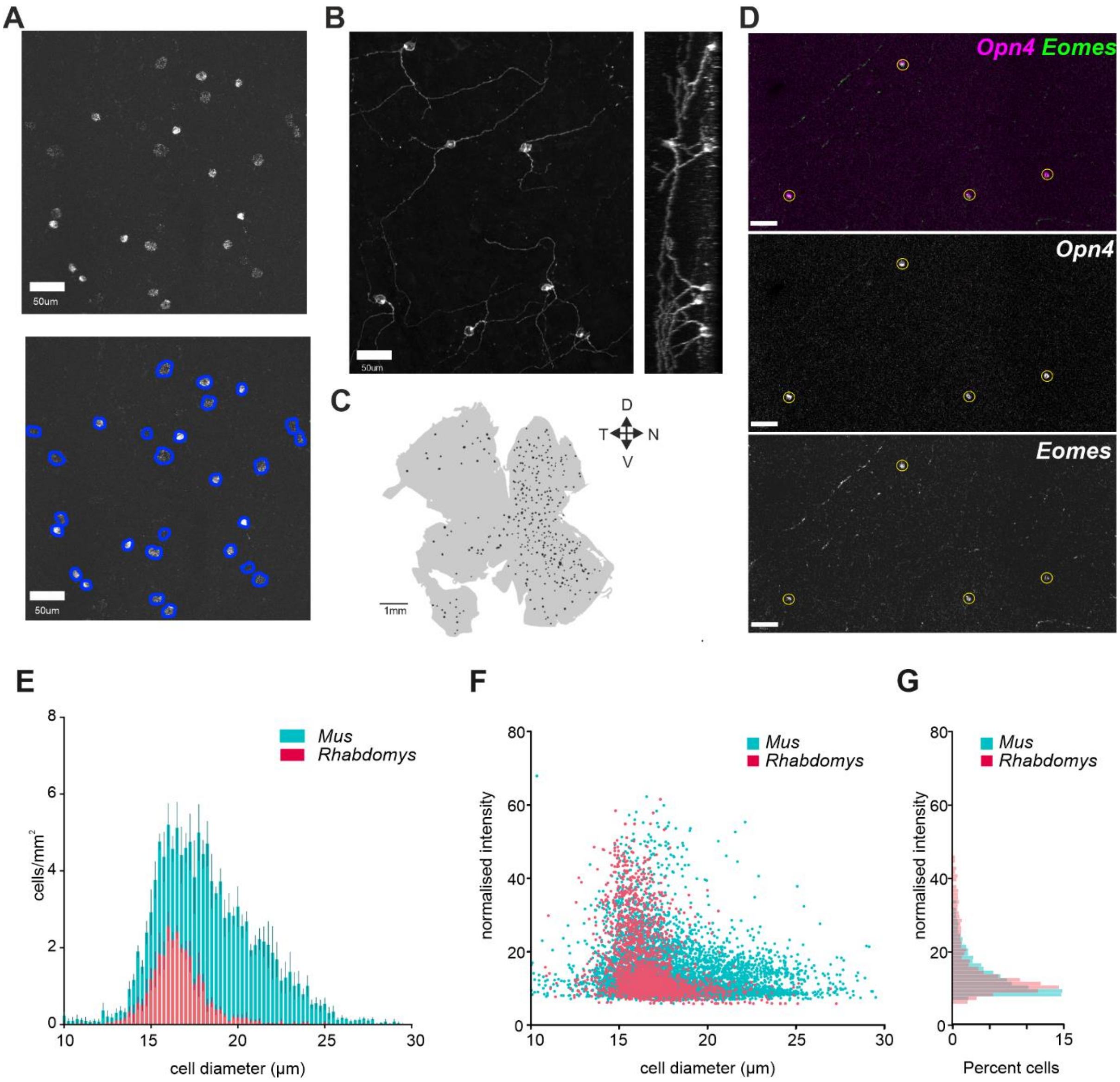

Figure S9. Automated cell identification. Related to figure 6.

A) Upper panel: representative HCR-FISH in *Mus* retinal wholemount, with probe for *Mus Opn4*. Scale bar = 50µm. Same wholemount, but with automated cell detection overlayed (blue edging). **B)** Immunohistochemistry for *Opn4* in *Rhabdomys* retina (Scale bar = 50µm). **D)** Retinal wholemount showing location of all Opn4 positive cell bodies. **C**) HCR-FISH in *Rhabdomys* retina with probes for *Rhabdomys Opn4* (magenta) and *Eomes (*green). Across whole retina 95.2% of *Opn4* positive cell bodies are *Eomes* positive. Lower panels show individual channels in greyscale. (Scale bars = 100µm). **E**) Histogram showing diameter of *Opn4* +ve cells/mm^2 in *Mus* (cyan) and *Rhabdomys* (red) retinal wholemounts (n=3; data show mean±SEM). **F**) Scatter plot showing diameter of *Opn4* +ve cells/mm^2 vs normalised signal intensity in *Mus* (cyan) and *Rhabdomys* (red) retinal wholemounts (n=3). **G)** Histogram showing normalised intensity of *Opn4* +ve cells in *Mus* (cyan) and *Rhabdomys* (red) retinal wholemounts (n=3 retinae; Kolmogorov-Smirnov test: P<0.001).

BC Type	*Rhab* 1	*Rhab* 2	*Mus* DropSeq	*Mus* ONC
BC1A	18.97	17.51	4.64	3.73
BC1B	2.61	1.83	3.37	1.40
BC2	4.82	5.01	2.37	4.36
BC3A	8.17	9.31	2.28	5.31
BC3B	13.91	15.38	3.48	8.96
BC4	6.41	6.53	1.69	3.38
BC5A	11.73	11.38	9.52	8.78
BC5B	4.42	3.98	2.04	5.63
BC5C	6.58	7.52	5.85	3.24
BC5D	2.24	1.92	2.35	2.30
BC6	3.91	3.88	7.24	11.59
BC7	8.17	7.92	7.49	5.33
BC8	0.66	0.74	0.52	1.96
BC9	2.12	2.21	0.80	2.03
RBC	5.27	4.87	46.35	31.97

Table S1. Bipolar cell frequencies across biological replicates of *Rhabdomys,* in comparison with *Mus.* Related to figure 5.
Rows show the percentage of *Rhabdomys* BCs mapping to each *Mus* BC type for two biological replicates, alongside previously published values for *Mus* BC classes [S2,S3].

BC Type	*Rhab* 1	*Rhab* 2	*Mus* DropSeq	*Mus* ONC
BC1A	20.03	18.41	8.65	5.48
BC1B	2.76	1.93	6.28	2.06
BC2	5.09	5.27	4.43	6.40
BC3A	8.63	9.79	4.24	7.81
BC3B	14.68	16.17	6.48	13.18
BC4	6.77	6.87	3.16	4.97
BC5A	12.39	11.96	17.75	12.91
BC5B	4.66	4.19	3.80	8.28
BC5C	6.95	7.91	10.9	4.76
BC5D	2.36	2.02	4.39	3.39
BC6	4.12	4.08	13.50	17.04
BC7	8.63	8.33	13.95	7.83
BC8	0.70	0.78	0.98	2.88
BC9	2.24	2.33	1.49	2.99

Table S2. Cone bipolar cell frequencies across biological replicates of *Rhabdomys,* in comparison with *Mus.* Related to figure 5.
As in table 1, but with rod BCs excluded. Thus, rows show the percentage of *Rhabdomys* cone BCs mapping to each *Mus* cone BC type for two biological replicates, alongside previously published values for *Mus* BC classes [S2,S3].

RGC Type	*Rhab* 1	*Rhab* 2	*Mus* 1	*Mus* 2	*Mus* 3
C1_W3L1	8.00	10.33	8.81	8.91	7.58
C2_W3D1	13.87	13.33	7.24	7.96	8.46
C3_FminiON	2.63	3.41	5.67	5.28	5.88
C4_FminiOFF	3.98	3.77	4.85	4.25	6.61
C5_JRGC	0.33	0.43	4.68	4.66	5.04
C6_W3B	7.47	7.18	3.80	4.92	4.24
C7	1.19	1.52	4.44	3.78	5.19
C8	2.11	2.76	2.98	3.57	3.76
C9_TRGC_novel	3.54	3.55	3.40	3.59	3.24
C10	1.05	1.05	3.71	3.19	3.16
C11	6.63	5.50	2.83	2.89	2.61
C12_ooDSGC_N	2.64	2.72	2.51	2.95	2.42
C13_W3L2	3.65	3.29	2.62	2.48	2.85
C14	0.87	0.88	2.64	2.60	2.17
C15	1.83	1.69	2.39	2.57	2.27
C16_ooDSGC_DV	3.48	3.21	3.11	2.25	2.00
C17_TRGC_S1	1.14	1.09	2.07	2.50	2.24
C18	0.94	0.97	2.11	2.79	1.84
C19	4.44	4.35	2.01	1.90	2.58
C20	1.99	1.47	1.80	2.00	2.08
C21_TRGC_S2	4.46	4.72	2.39	1.37	2.35
C22_MX	0.24	0.33	1.19	1.90	1.74
C23_W3D2	4.11	3.37	2.01	1.22	2.07
C24	3.93	3.53	2.05	1.58	1.25
C25	2.07	1.81	1.85	1.75	1.07
C26	2.28	1.83	1.73	1.61	1.23
C27	1.17	1.07	1.72	1.57	1.25
C28_FmidiOFF	0.49	0.54	1.20	1.63	1.36
C29	2.64	2.73	1.35	1.63	1.15
C30_W3D3	1.42	1.83	1.45	1.03	1.75
C31_M2	0.18	0.12	0.91	1.58	1.01
C32_FRGC_novel	0.53	0.58	1.51	1.11	0.98
C33_M1a	0.03	0.08	0.79	0.91	0.96
C34	1.14	1.01	1.04	0.63	1.08
C35	1.32	1.50	0.86	0.97	0.75
C36	0.21	0.12	0.81	0.64	0.61
C37	0.23	0.24	0.85	0.67	0.38
C38_FmidiON	0.17	0.24	0.56	0.65	0.50
C39	1.00	1.06	0.57	0.37	0.80
C40_M1b	0.02	0.01	0.26	0.56	0.52
C41_AlphaONT	0.06	0.15	0.31	0.42	0.30
C42_AlphaOFFS	0.31	0.24	0.41	0.34	0.24
C43_AlphaONS_M4	0.08	0.12	0.25	0.42	0.18
C44	0.10	0.21	0.10	0.20	0.16
C45_AlphaOFFT	0.03	0.07	0.16	0.20	0.08

Table S3. Retinal ganglion cell frequencies across biological replicates of *Rhabdomys,* in comparison with *Mus.* Related to figure 6.
Each row shows the percentage of RGCs mapping to each *Mus* RGC type for two biological replicates of *Rhabdomys,* and three biological replicates of *Mus* [S3].

Supplemental References

S1. Prusky, G.T., West, P.W., and Douglas, R.M. (2000). Behavioral assessment of visual acuity in mice and rats. Vision Res 40, 2201-2209. 10.1016/s0042-6989(00)00081-x.

S2. Shekhar, K., et al. (2016). Comprehensive Classification of Retinal Bipolar Neurons by Single-Cell Transcriptomics. Cell 166, 1308-1323 e1330. 10.1016/j.cell.2016.07.054.

S3. Tran, N.M., et al. (2019). Single-Cell Profiles of Retinal Ganglion Cells Differing in Resilience to Injury Reveal Neuroprotective Genes. Neuron 104, 1039-1055 e1012. 10.1016/j.neuron.2019.11.006.
